# Methanol-Driven Oxidative Rearrangement of Biogenic Furans – Enzyme Cascades vs. Photobiocatalysis

**DOI:** 10.3389/fchem.2021.635883

**Published:** 2021-04-07

**Authors:** Christina Jäger, Cloé Bruneau, Philip K. Wagner, Martin H. G. Prechtl, Jan Deska

**Affiliations:** ^1^ Department of Chemistry, Aalto University, Espoo, Finland; ^2^ Department of Chemistry, University of Cologne, Cologne, Germany; ^3^ Instituto Superior Técnico, University of Lisbon, Lisboa, Portugal

**Keywords:** ring expansion, oxygenation, aerobic, furans, enzymatic, visible light

## Abstract

The oxidative ring expansion of bio-derived furfuryl alcohols to densely functionalized six-membered O-heterocycles represents an attractive strategy in the growing network of valorization routes to synthetic building blocks out of the lignocellulosic biorefinery feed. In this study, two scenarios for the biocatalytic Achmatowicz-type rearrangement using methanol as terminal sacrificial reagent have been evaluated, comparing multienzymatic cascade designs with a photo-bio-coupled activation pathway.

## Introduction

**FIGURE 1:**
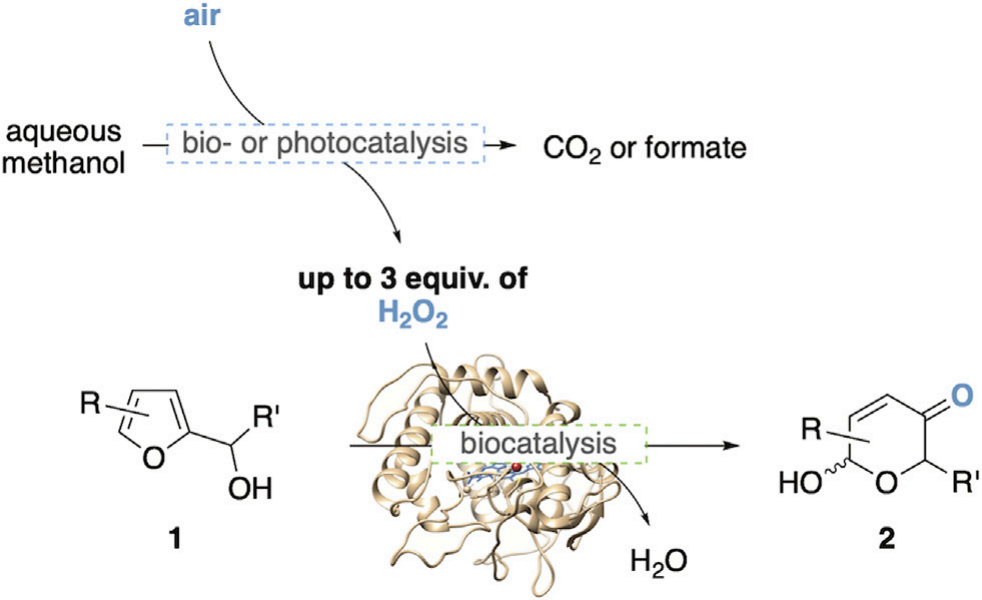
Enzymatic or photocatalytic O_2_-activation as tools for methanol-driven Achmatowicz-type ring expansions.

Since its discovery in the early 1970’s (Achmatowicz et al., 1971; Lefebvre, 1972), the Achmatowicz rearrangement has gained substantial recognition by the synthetic community (Deska et al., 2015) and has found versatile applications in the preparation of complex heterocyclic target structures (Ghosh and Brindisi, 2016; Blume et al., 2016; Naapuri et al., 2017). Utilizing furfuryl alcohols as the basic starting point of the ring expansion strategy to substituted hydroxypyranones, in recent years this oxidative rearrangement has attracted substantial interest also as a potentially valuable tool for future value chains based on lignocellulosic biomass, where the furfural platform represents a key component in the endeavor to create value chains that are independent on fossil resources (Mariscal, 2016; Kabbour and Luque, 2020). Since traditional Achmatowicz rearrangements are conducted in presence of stoichiometric oxidants such as *N*-bromosuccinimide or peracids, developments toward modern catalytic versions of this reaction have aimed to substitute these less sustainable chemical oxidants by aerobic activation pathways. In particular, enzyme catalysis has significantly expanded the furan oxidation toolbox, and a series of biocatalytic protocols utilizing enzyme-mediator systems have been reported, including couples based on H_2_O_2_/haloperoxidase/halide (Fernández-Fuego et al., 2016), air/laccase/TEMPO (Asta et al., 2013) and H_2_O_2_/lipase/acetate (Blume et al., 2018). As part of our group’s campaign to design novel biocatalytic applications to mimic classical non-natural synthetic-organic reactions, we successfully developed a non-mediated, enzymatic Achmatowicz methodology exploiting the direct oxygenation of furans through commercial chloroperoxidase from *C. fumago* (Thiel et al., 2014). Here, the oxygen-transfer biocatalyst is supplemented by glucose oxidase (GOx) to provide the necessary hydrogen peroxide via reduction of air. Although this protocol has been proven to be highly effective, and has been implemented into synthetic applications (Blume et al., 2016) and biocatalytic cascades (Liu et al., 2018), we have pursued the search for alternative aerobic activation routes in order to substitute the glucose, on one side to create complementary sacrificial agents for the design of more complex biocascades, but also due to the somewhat poor atom-economy of the glucose. Among the various potential reductants to drive peroxidase-catalyzed reactions, methanol stands out as most attractive reagent as it offers the highest hydrogen density of common, enzyme-compatible small molecules and as it can theoretically provide up to three equivalents of hydrogen peroxide when oxidized all the way to carbon dioxide (Kara et al., 2015). Following the recent literature, two complementary approaches caught our attention which were evaluated and compared in this study (Figure 1). In a first design, the chloroperoxidase-catalyzed furan oxidation was coupled to C1-active biocatalysts to arrive at a multienzymatic network for the methanol-fueled Achmatowicz reaction. For the second approach, the methanol-activating enzyme modules were replaced by a water-soluble photocatalyst (Yuan et al., 2020) to create a visible light-driven process for the synthesis of the synthetically valuable, functionalized pyranone products.

## Results and Discussion

Our investigations toward a methanol-driven biocatalytic Achmatowicz protocol commenced with an in-depth screening of parameters affecting the oxidase/peroxidase-coupled rearrangement of model substrate **1a**. Following our previous work on enzyme-metal-coupled methanol reforming (Heim et al., 2015), alcohol oxidase from *P. pastoris* was chosen as hydrogen peroxide generating module (Couderc and Baratti, 1980), and supplemented to the *C. fumago* chloroperoxidase. At 10 mM concentration of furfuryl alcohol **1a**, stepwise increase of the oxidase loading resulted in increased conversion rates (Table 1, entries 1 & 2), however, at excessive concentrations of the methanol-active catalyst, a slight decline was observed (Table 1, entry 3). Similarly, very high concentrations of methanol turned out to be disadvantageous to the overall system (Table 1, entry 5). A substantial improvement could be achieved by lowering of the substrate concentration where up to 76% conversion of alcohol **1a** where detected (Table 1, entries 6 & 7). Methanol oxidation by the fungal oxidase results in formation of formaldehyde as primary product alongside the hydrogen peroxide, and the aldehyde was believed to pose a risk as reactive and potentially inactivating species. Gratifyingly, addition of formaldehyde dismutase (Kato et al., 1983) to the reaction mixture resulted in a significant increase in conversion (Table 1, entry 8). Here, the dismutase does not only decompose the aldehyde intermediate but, as the biocatalytic disproportionation provides more methanol, the process is rendered more effective and close to full consumption of **1a** can be achieved at lower initial methanol concentrations (Kara et al., 2015).

**TABLE 1:** Optimization of the oxidase/peroxidase-coupled Achmatowicz rearrangement. 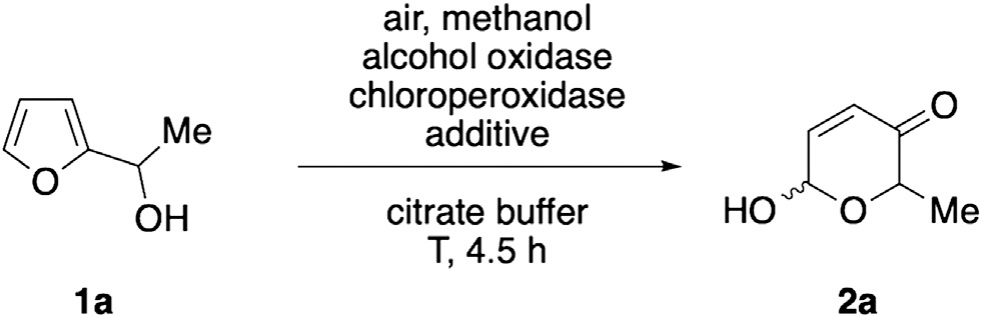

Entry	Furfuryl alcohol 1a (mM)	Alcohol oxidase (U/mmol 1a)	methanol (equiv.)	T (°C)	Additive	Conversion (%)
1	10	10	5.0	30	None	51
2	10	30	5.0	30	None	64
3	10	100	5.0	30	None	61
4	10	30	1.2	30	None	39
5	10	30	50	30	None	9
6	5	60	5.0	30	None	75
7	5	60	5.0	35	None	76
8	5	60	2.5	35	Formaldehyde dismutase	88

The biocatalytic method combining alcohol oxidase, formaldehyde dismutase and chloroperoxidase was subsequently evaluated with regard to its substrate scope and the ability to oxidatively convert different furan substrates. To our delight, a wide range of furfuryl alcohols was accepted and after incubation over night, good to excellent conversions were observed for nearly all alkyl-substituted secondary alcohols. Particularly bulky substrates that demand a moderately slow hydrogen-peroxide supply (Thiel et al., 2018) performed well with the methanol-activating biocascade and oxidation reached full conversion after 24 h (Table 2, entries 2–4). In contrast, the chloromethyl substituted **1h** and the core-methylated **1i** reacted more sluggishly and exhibited only conversions around 70% even after long incubation times (Table 2, entries 8 & 9). The benzylic alcohol **1j**, that was inactive under the previously reported glucose-driven system, also remained unaffected under the herein described alternative activation protocol (Table 2, entry 10). Dehydrogenation of the heterobenzylic substrates (**1a**-**1j)** through alcohol oxidase under formation of the corresponding ketones was not observed, underlining the good fit of the different components in this multienzymatic cocktail.

**TABLE 2:** Substrate scope of the triple-enzymatic Achmatowicz oxidation. 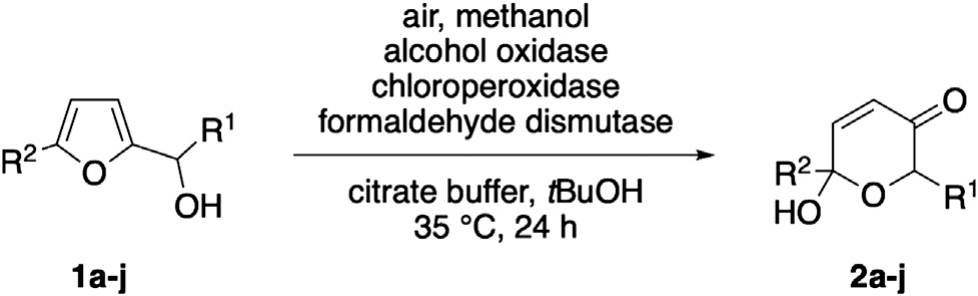

Entry	Furfuryl alcohol	R^1^	R^2^	Conversion (%)
1	**1a**	Me	H	94
2	**1b**	*t*Bu	H	99
3	**1c**	*i*Bu	H	99
4	**1d**	*i*Pr	H	99
5	**1e**	*n*Pr	H	97
6	**1f**	Et	H	88
7	**1g**	*n*Hex	H	99
8	**1h**	CH_2_Cl	H	71
9	**1i**	Me	Me	69
10	**1j**	Ph	H	<5

**FIGURE 2:**
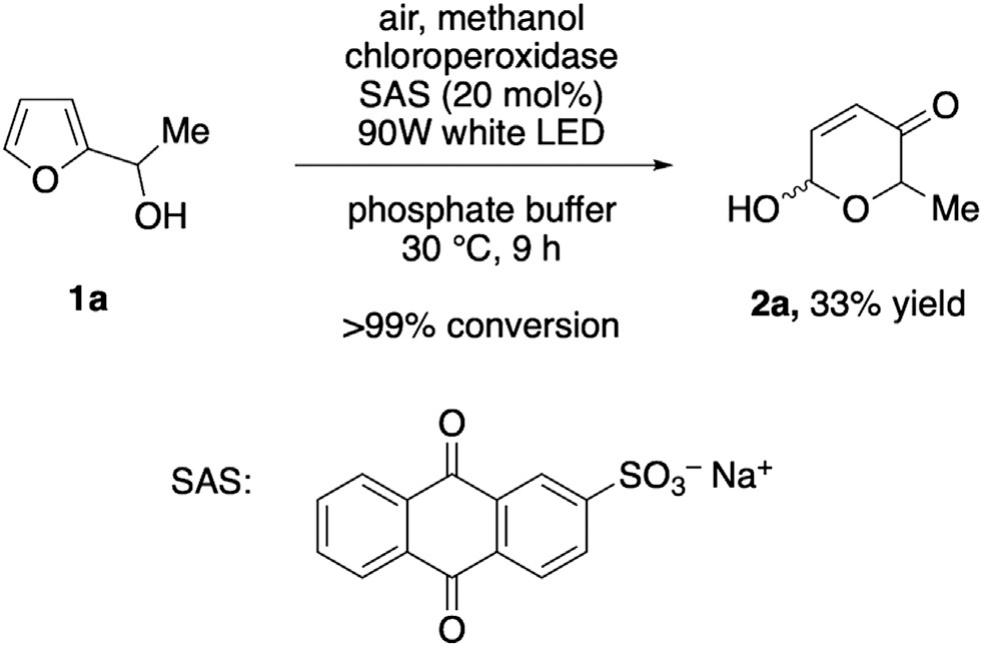
Photoenzymatic Achmatowicz-type ring expansion.

Inspired by the recent reports on water-soluble anthraquinones as photoactive additives for oxygenative enzyme reactions (Zhang et al., 2017; Yuan et al., 2020), we next replaced the oxidase/dismutase module by sodium anthraquinone-2-sulfonate (SAS) in order to create a photo-biocatalytic scenario as alternative approach, where complete oxidation of methanol to CO_2_ would offer an even more elegant route. After extensive aeriation, the solutions with the yellow dye were irradiated with white LEDs. However, despite thorough optimization efforts, this alternative pathway proved to be very challenging. In presence of 20 mol% SAS, full conversion of the model substrate **1a** was achieved within 9 h, yet, the product selectivity significantly lagged behind the dark, enzyme-coupled protocol (Figure 2). Based on the observation that only minor traces of the corresponding ketone could be detected, we believe that the initial Achmatowicz rearrangement is in fact supported by the SAS/CPO couple and that the photocatalyst remains active throughout the irradiation period (see also Supplementary Figure S2). However, the dense functionalization of **2a** also renders the product vulnerable for subsequent oxidation events, both through further dehydrogenation of the hemiacetal position by SAS and through unspecific oxygenation by other reactive oxygen species that are present in the SAS-mediated environment (Zhang et al., 2017).

**FIGURE 3:**
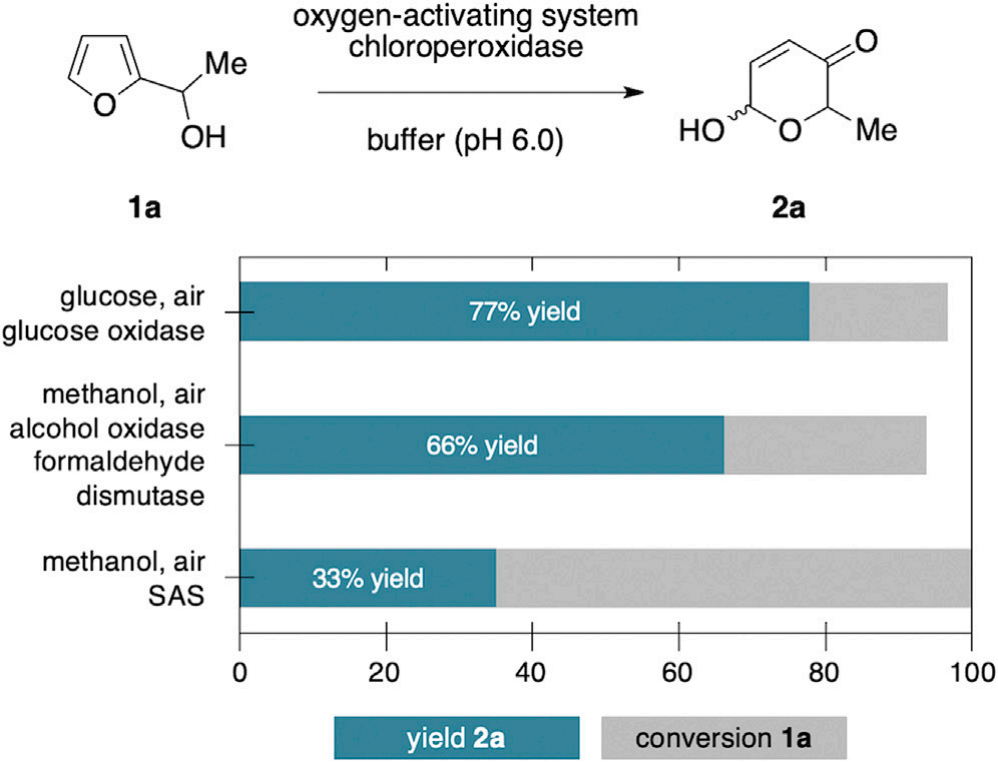
Comparison of product selectivity between the different enzymatic Achmatowicz protocols.

As all Achmatowicz reactions provide densely functionalized pyranone products (**2**) that carry multiple reactive groups, very high yields pose a general challenge for any methodology aiming for selective furan oxidation protocols. One way to address this issue specifically within enzymatic catalysis is the creation of biocatalyic cascades where the pyranone is further telescoped *in situ* to a more stable final product (Liu et al., 2018). Nevertheless, to shed light on the synthetic value of the different methods beyond the initial catalytic optimization, preparative yields of the product **2a** need to be considered. Here, the purely biocatalytic methanol-driven protocol that provides 66% yield of **2a** still compares well with the previously developed GOx/CPO-system (77%). Future studies that implement the methanol-activation protocol in more complex cascades will eventually show if this novel tool has the true potential in synthetic settings. In contrast, the photo-biocatalytic approach clearly lacks the necessary selectivity and even if no clear side product profile could be detected, the final yield of 33% is a very strong indicator that the high reactivity of the photoexcited anthraquinone catalyst poses an inevitable challenge in the context of Achmatowicz oxidations (Figure 3).

**FIGURE 4:**
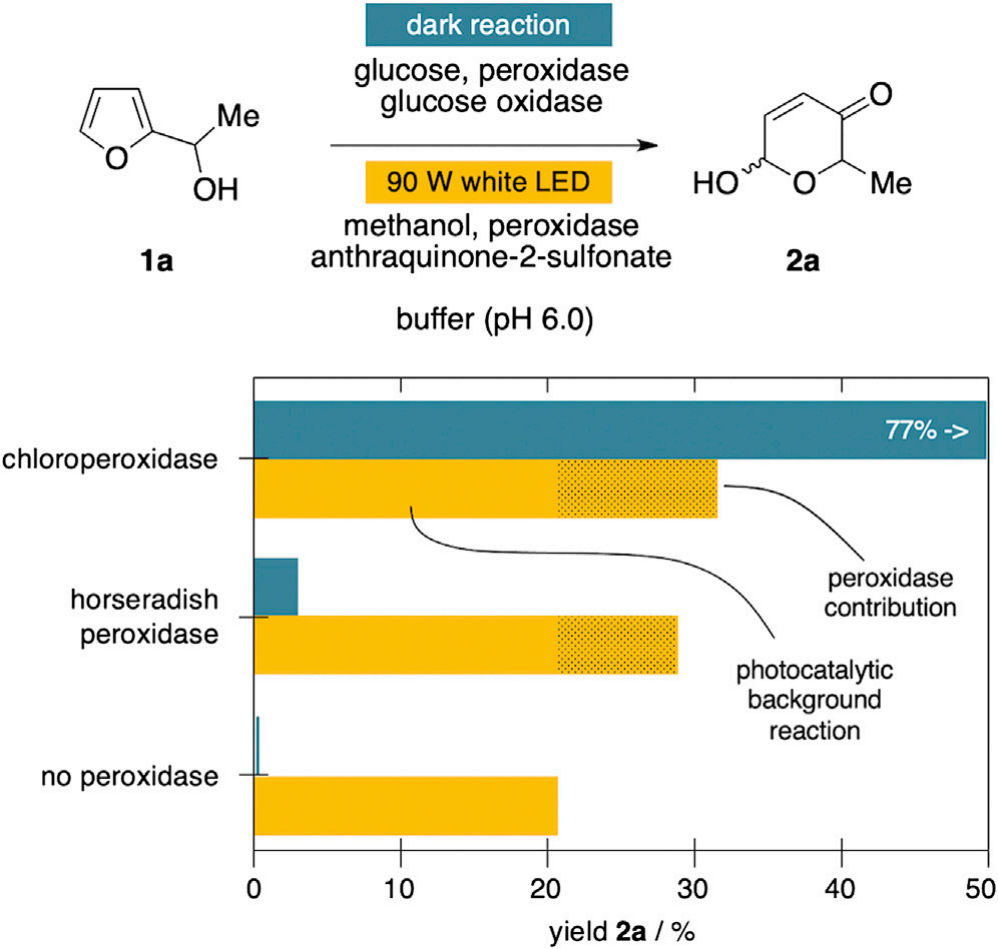
Influence of sodium anthraquinone-2-sulfonate on peroxidase-mediated ring expansions.

We recently discovered that also horseradish peroxidase (HRP) exhibits promiscuous behavior and can act as efficient catalyst in oxidative, non-natural transformations (Jäger and Deska, 2021). Despite the fact that HRP performed poorly in previous investigations on Achmatowicz rearrangements, we decided to revisit the enzyme as part of this study. To our surprise, also in presence of horseradish peroxidase instead of the haloperoxidase, substantial conversion of **1a** was recorded, exceeding the reactivity of GOx/HRP couple considerably (Figure 4). A control reaction in absence of any peroxidase did reveal an underlying oxidation pathway triggered through the non-H_2_O_2_ reactive oxygen species from the anthraquinone excitation (Zhang et al., 2017), again underlining the poor match of the SAS system in the Achmatowicz context. Nevertheless, even after elimination of the background conversion of **1a** to **2a** by SAS alone, it is worth having a closer look on the discrepancy between the horseradish peroxidase's contribution to the yield of **2a** under dark and light conditions. In direct comparison, HRP can only achieve less than 5% of the overall yield relative to the CPO benchmark system when fueled by glucose oxidase. In stark contrast, in the SAS-coupled system the histidine-ligated horseradish peroxidase contributes to the formation of **2a** much more significantly, reaching 58% of the cysteine-ligated CPO’s performance (see peroxidase contribution in Figure 4). Even though it has become obvious that the Achmatowicz reaction is incompatible with the SAS-peroxidase approach due to the high reactivity of the ring expansion products, this small but measurable effect related to otherwise inactive HRP could be of interest in follow-up studies. The general observation of enhanced reactivities of the horseradish peroxidase under those photocatalytic conditions warrants a closer look as part of future investigations on less side-reaction-prone substrates, and whether or not photo-boosting of oxygenating properties of these classical heme enzymes could be exploited.

## Conclusion

In summary, we have investigated two complementary pathways for the enzymatic ring rearrangement of biogenic furfuryl alcohols based on methanol-driven hydrogen peroxide generation modules a) via a multi-enzymatic route and b) in a photobio-coupled scenario. A triple-enzymatic method was developed exploiting alcohol oxidase and formaldehyde dismutase as effective H_2_O_2_-producing system that enabled the aerobic oxidative rearrangement of a wide range of furanoic building blocks that can be directly derived from lignocellulosic biomass. The photoenzymatic design on the other hand exposed some challenges as the reactive oxygen species generated by a water-soluble anthraquinone photocatalyst rendered the process less selective and did not proceed with high product yields. Nevertheless, our study also revealed an interesting interplay between the dye and the designated heme-based oxygenation biocatalysts which will be further investigated in future studies.

## Materials and Methods

### General Remarks

Commercially available reagents were used without further purification. Furfuryl alcohols were prepared according to literature procedures through addition of Grignard reagents to furfural. All biocatalytic experiments utilized the following enzymes: horseradish peroxidase (173 U/mg), chloroperoxidase (*Caldariomyces fumago*, 37,332 U/mL), alcohol oxidase (*Pichia pastoris*, 1,196 U/mL), formaldehyde dismutase (*Pseudomonas putida*). The photocatalytic experiments were conducted using an array of three 30W LED floodlights (Shining Star, 230V, 2,400 lm, 6,500 K) surrounding the reaction vessels. An overhead household fan was attached to maintain ambient temperature (for the setup, see also, Supplementary Figure S1 in the Supplementary Materials). Silica gel from Merck (Millipore 60, 40–60 μm, 240–400 mesh) was used for column chromatography and silica pad filtrations. Reactions were monitored via thin layer chromatography (TLC) using precoated silica gel plates from Machery-Nagel (TLC Silica gel 60 F_254_). The spots were identified using irradiation with UV light and a staining solution (basic potassium permanganate solution). ^1^H and ^13^C NMR spectra were measured on Bruker Avance NEO 400 or Bruker Avance 300 spectrometers, respectively, at 20°C. The chemical shifts are reported in ppm related to the signal of residual solvent of CDCl_3_ (^1^H: (CDCl_3_) = 7.26 ppm, ^13^C: (CDCl_3_) = 77.2 ppm). Infrared-spectra were recorded on a Shimadzu IRAffinity-1 FT-IR-spectrometer, absorption bands are reported in wave numbers [cm^−1^].

### Representative Procedure for the Triple-Enzymatic Achmatowicz Rearrangement

Furfuryl alcohol 1a (0.05 mmol) was dissolved in 10 ml citrate buffer (pH 6.0, 100 mM) containing 10 vol% *t*BuOH. Next, 25.1 μl alcohol oxidase (*P. pastoris*), 26.8 μl chloroperoxidase (*C. fumago*), 2.0 mg formaldehyde dismutase (*P. putida*) and 5 μl MeOH were added. The reaction mixture was incubated at 35°C and 180 rpm. L-Methionine was added after 3.5 h and the aqueous mixture was extracted with EtOAc (4×). The combined organic layers were dried over Na_2_SO_4_, filtered and the solvent was removed under reduced pressure. 6-Hydroxy-2-methyl-2*H*-pyran-3(6*H*)-one (2a) was obtained with 66% yield, as determined by ^1^H-NMR spectroscopy (against dimethyl sulfone as internal standard).

### Representative Procedure for the Photoenzymatic Achmatowicz Rearrangement

Furfuryl alcohol 1a (0.1 mmol) was added to 4 ml solution of sodium anthraquinone-2-sulfonate (5 mM in phosphate buffer pH 6.0, 60 mM) and 6 ml phosphate buffer (pH 6.0, 60 mM) containing 0.5% MeOH referred to the total reaction volume of 10 ml. To this mixture 2.67 μl chloroperoxidase (*C. fumago*) was added and the reaction was irradiated (90 W white light) at 30°C under oxygen atmosphere. L-Methionine was added after 9 h, and the reaction mixture was extracted with EtOAc (4×). The combined organic layers were dried over Na_2_SO_4_, filtered and the solvent was removed under reduced pressure. 6-Hydroxy-2-methyl-2*H*-pyran-3(6*H*)-one (2a) was obtained with 32% yield, as determined by ^1^H-NMR spectroscopy (against dimethyl sulfone as internal standard).

## Data Availability

The original contributions presented in the study are included in the article/Supplementary Material, further inquiries can be directed to the corresponding author.
