# Supplementary material for: Methanol-Driven Oxidative Rearrangement of Biogenic Furans – Enzyme Cascades vs. Photobiocatalysis
Source: Front Chem. 2021 Apr 7;9:635883. doi: 10.3389/fchem.2021.635883 (PMC8058437; doi:10.3389/fchem.2021.635883)
Supplement: Supplementary file 1 [file datasheet1.pdf]

Supplementary Material

Methanol-driven Oxidative Rearrangement of Biogenic Furans – Enzyme Cascades vs Photobiocatalysis

Christina Jäger,[a] Cloé Bruneau,[a] Philip K. Wagner,[a,b] Martin H. G. Prechtl,[b,c] Jan Deska[a*]

[a] Department of Chemistry, Aalto University, 02150 Espoo, Finland

[b] Department of Chemistry, University of Cologne, 50939 Cologne, Germany

[c] Instituto Superior Técnico, University of Lisbon, 1049-001 Lisboa, Portugal

*Correspondence: jan.deska@aalto.fi

General remarks

Commercially available reagents were used without further purification. Furfuryl alcohols were prepared according to literature procedures through addition of Grignard reagents to furfural. All biocatalytic experiments utilized the following enzymes: horseradish peroxidase (173 U/mg), chloroperoxidase (*Caldariomyces fumago*, 37332 U/mL), alcohol oxidase (*Pichia pastoris*, 1196 U/mL), formaldehyde dismutase (*Pseudomonas putida*). The photocatalytic experiments were conducted using an array of three 30W LED floodlights (Shining Star, 230V, 2400 lm, 6500 K) surrounding the reaction vessels. An overhead household fan was attached to maintain ambient temperature (see setup below in Figure S1). Silica gel from Merck (Millipore 60, 40-60 µm, 240-400 mesh) was used for column chromatography and silica pad filtrations. Reactions were monitored via thin layer chromatography (TLC) using precoated silica gel plates from Machery-

Nagel (TLC Silica gel 60 F_{254}). The spots were identified using irradiation with UV light and a staining solution (basic potassium permanganate solution). ^{1}H and ^{13}C NMR spectra were measured on Bruker Avance NEO 400 or Bruker Avance 300 spectrometers, respectively, at 20 °C. The chemical shifts are reported in ppm related to the signal of residual solvent of $CDCl_3$ (^{1}H: ($CDCl_3$) = 7.26 ppm, ^{13}C: ($CDCl_3$) = 77.2 ppm). Infrared-spectra were recorded on a Shimadzu IRAffinity-1 FT-IR-spectrometer, absorption bands are reported in wave numbers [cm^{-1}].

Representative procedure for the triple-enzymatic Achmatowicz rearrangement

0.05 mmol furfuryl alcohol was dissolved in 10mL citrate buffer (pH 6.0, 100 mM) containing 10 vol% *t*-BuOH. Next, 25.1 µL alcohol oxidase (*P. pastoris*), 26.8 µL chloroperoxidase (*C. fumago*), 2.0 mg formaldehyde dismutase (*P. putida*) and 5 µL MeOH were added. The reaction mixture was incubated at 35 °C and 180 rpm. L-Methionin was added after 3.5 h and the aqueous mixture was extracted 4x with EtOAc. The organic phases were dried over Na_2SO_4, filtered and the solvent was removed under reduced pressure. 6-Hydroxy-2-methyl-2*H*-pyran-3(6*H*)-one was obtained with 66 % yield, as determined by ^{1}H-NMR spectroscopy (against dimethyl sulfone as internal standard).

Representative procedure for the photoenzymatic Achmatowicz rearrangement

0.1 mmol furfuryl alcohol were added to 4 mL solution of sodium anthraquinone-2-sulfonate (5 mM in phosphate buffer pH 6.0, 60 mM) and 6 mL phosphate buffer (pH 6.0, 60 mM) containing 0.5 % MeOH referred to the total reaction volume of 10 mL. To this mixture 2.67 µL chloroperoxidase (*C. fumago*) was added and the reaction was irradiated (90 W white light) at 30 °C under oxygen atmosphere. L-Methionine was added after 9 h the reaction mixture was extracted 4x with EtOAc. The organic phases were dried over Na_2SO_4, filtered and the solvent was removed under reduced pressure. 6-Hydroxy-2-methyl-2*H*-pyran-3(6*H*)-one was obtained with 32 % yield, as determined by ^{1}H-NMR spectroscopy (against dimethyl sulfone as internal standard).

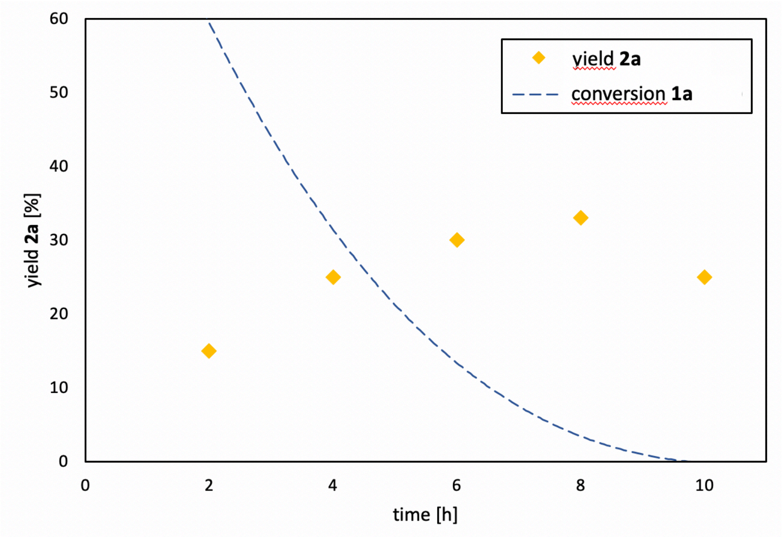

SUPPLEMENTARY FIGURE S1 | Product formation under photo-biocatalytic conditions.

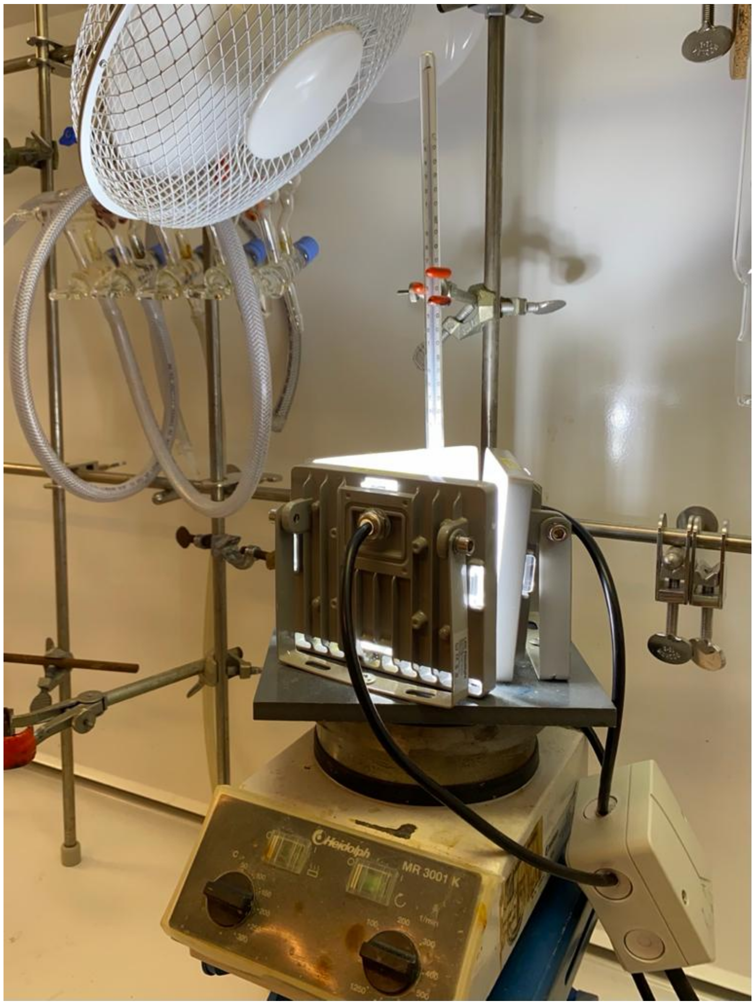

SUPPLEMENTARY FIGURE S2 | Photocatalysis setup.

Analytical data of the products

6-Hydroxy-2-methyl-2*H*-pyran-3(6*H*)-one (**2a**)

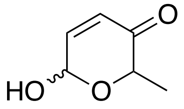

2a

R*f* (cyclohexane/ethyl acetate, 2/1): 0.25. **α-2a**: **^{1}H-NMR** (300 MHz, $CDCl_3$): δ [ppm] = 6.89 (dd, ^{3}J = 10.2 Hz, ^{3}J = 3.2 Hz, 1H), 6.10 (d, ^{3}J = 10.2 Hz, 1H), 5.63 (m, 1H), 4.71 (q, ^{3}J = 6.7 Hz, 1H), 3.53 (d, ^{3}J = 5.0 Hz, 1H), 1.38 (d, ^{3}J = 6.7 Hz, 3H). **^{13}C-NMR** (75 MHz, $CDCl_3$): δ [ppm] = 197.0, 144.5, 127.3, 87.7, 70.4, 15.3. **β-2a**: **^{1}H-NMR** (300 MHz, $CDCl_3$): δ [ppm] = 6.94 (d, ^{3}J = 10.2 Hz, 1H), 6.15 (d, ^{3}J = 10.2 Hz, 1H), 5.67 (d, ^{3}J = 7.2 Hz, 1H), 4.23 (1H, m), 3.87 (d, ^{3}J = 7.2 Hz, 1H), 1.45 (d, ^{3}J = 6.7 Hz, 3H). **^{13}C-NMR** (75 MHz, $CDCl_3$): δ [ppm] = 196.5, 148.1, 128.6, 91.0, 75.3, 16.2. **FT-IR** (neat, ATR): ν [cm^{-1}] = 3294 (br), 3051 (w), 2987 (w), 1676 (s), 1435 (m), 1371 (m), 1334 (w), 1273 (w), 1232 (m), 1143 (m), 1109 (m), 1091 (m), 1031 (s), 937 (m), 900 (m), 808 (m), 690 (m).

6-Hydroxy-2-*tert*-butyl-2*H*-pyran-3(6*H*)-one (**2b**)

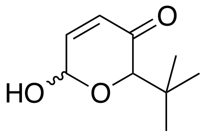

2b

R*f* (cyclohexane/ethyl acetate, 2/1): 0.35. **α-2b**: **^{1}H-NMR** (300 MHz, $CDCl_3$): δ [ppm] = 6.84 (dd, ^{3}J = 10.2 Hz, ^{3}J = 3.2 Hz, 1H), 6.04 (d, ^{3}J = 10.2 Hz, 1H), 5.66 (d, ^{3}J = 3.2 Hz, 1H), 4.21 (s, 1H), 3.48 (br s, 1H), 1.06 (s, 9H). **^{13}C-NMR** (75 MHz, $CDCl_3$): δ [ppm] = 196.8, 143.4, 128.8, 87.9, 80.2, 34.9, 26.3. **β-2a** (selected signals): **^{1}H-NMR** (300 MHz, $CDCl_3$): δ [ppm] = 6.89-6.85 (m, 1H), 6.10- 6.06 (m, 1H), 5.60 (m, 1H), 3.70 (s, 1H), 1.09 (s, 9H).**^{13}C-NMR** (75 MHz, $CDCl_3$): δ [ppm] = 196.1, 147.1, 130.4, 91.8, 85.1. **FT-IR** (neat, ATR): ν [cm^{-1}] = 3371 (br), 2958 (m), 2872 (w), 1726 (m), 1681 (s), 1633 (w), 1463 (w), 1363 (m), 1300 (m), 1234 (m), 1182 (m), 1089 (m), 1026 (s), 1004 (s), 968 (m), 900 (m), 821 (w), 746 (m).

6-Hydroxy-2-isobutyl-2*H*-pyran-3(6*H*)-one (**2c**)

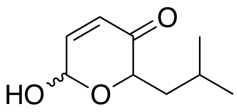

2c

R*f* (cyclohexane/ethyl acetate, 2/1): 0.43. **α-2c**: **^{1}H-NMR** (300 MHz, $CDCl_3$): δ [ppm] = 6.88 (dd, ^3J = 10.1 Hz, ^3J = 3.3 Hz, 1H), 6.10 (d, ^3J = 10.3 Hz, 1H), 5.66- 5.63 (m, 1H), 4.60 (dd, ^3J = 9.7 Hz, ^3J = 3.4 Hz, 1H), 3.04 (br s, 1H), 1.86-1.53 (m, 3H), 0.94 (m, 6H). **^{13}C-NMR** (75 MHz, $CDCl_3$): δ [ppm] = 193.1, 143.9, 127.8, 87.4, 72.5, 38.2, 24.2, 23.4, 21.2). **β-2c** (selected signals): **^{1}H-NMR** (300 MHz, $CDCl_3$): δ [ppm] = 6.94-6.89 (m, 1H, H-2), 6.17- 6.12 (m, 1H), 5.66-5.63 (m, 1H), 4.15-4.09 (m, 1H), 3.26 (br s, 1H), 1.86-1.53 (m, 3H), 0.94 (t, ^3J = 6.1 Hz, 6H). **^{13}C-NMR** (75 MHz, $CDCl_3$): δ [ppm] = 147.1, 129.0, 90.9, 82.3, 39.3. **FT-IR** (neat, ATR): ν [cm^{-1}] = 3307 (br), 2954 (m), 2873 (m), 1672 (m), 1629 (w), 1471 (m), 1388 (w), 1371 (m), 1317 (w), 1276 (m), 1226 (m), 1157 (m), 1091 (s), 1020 (s), 975 (m), 898 (m), 827 (w), 786 (m), 702 (m).

6-Hydroxy-2-(2-propyl)-2*H*-pyran-3(6*H*)-one (**2d**)

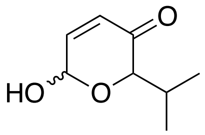

2d

R*f* (cyclohexane/ethyl acetate, 2/1): 0.32. **α-2d**: **^{1}H-NMR** (300 MHz, $CDCl_3$): δ [ppm] = 6.93 (dd, ^3J = 10.2 Hz, ^3J = 3.4 Hz, 1H), 6.12 (d, ^3J = 10.2 Hz, 1H), 5.71-5.65 (m, 1H), 4.43 (d, ^3J = 3.4 Hz, 1H), 3.15-3.10 (m, 1H), 2.52-2.41 (m, 1H), 1.05 (d, ^3J = 7.1 Hz, 3H), 0.90 (d, ^3J = 6.9 Hz, 3H). **^{13}C-NMR** (75 MHz, $CDCl_3$): δ [ppm] = 196.2, 144.2, 128.1, 87.7, 77.3, 28.6, 19.0, 16.2. **β-2d** (selected signals): **^{1}H-NMR** (300 MHz, $CDCl_3$): δ [ppm] = 6.14 (dd, ^3J = 10.3 Hz, ^3J = 1.5 Hz, 1H), 3.93 (dd, ^3J = 3.4 Hz, ^3J = 1.3 Hz, 1H), 3.43-3.37 (m, 1H), 0.96 (d, ^3J = 6.8 Hz, 3H). **^{13}C-NMR** (75 MHz, $CDCl_3$): δ [ppm] = 148.0, 129.5, 91.2, 83.1, 28.9, 19.1, 16.5. **FT-IR** (neat, ATR): ν [cm^{-1}] = 3385 (br), 2966 (w), 1681 (m), 1463 (w), 1369 (m), 1273 (w), 1230 (w), 1153 (w), 1082 (m), 1016 (s), 946 (m), 885 (w), 756 (m), 680 (w).

6-Hydroxy-2-propyl-2*H*-pyran-3(6*H*)-one (**2e**)

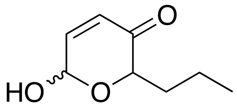

2e

R*f* (cyclohexane/ethyl acetate, 2/1): 0.29. **α-2e**: **^{1}H-NMR** (300 MHz, $CDCl_3$): δ [ppm] = 6.92 (dd, ^{3}J = 10.1 Hz, ^{3}J = 3.2 Hz, 1H), 6.15 (d, ^{3}J = 10.2 Hz, 1H), 5.68-5.64 (m, 1H), 4.58 (dd, ^{3}J = 8.1 Hz, ^{3}J = 3.9 Hz, 1H), 3.17 (br s, 1H), 2.10-1.39 (m, 4H), 0.96 (t, ^{3}J = 7.4 Hz ,3H). **^{13}C-NMR** (75 MHz, $CDCl_3$): δ [ppm] = 194.8, 144.3, 127.6, 87.6, 74.0, 31.6, 18.2, 13.8. **β-2e** (selected signals): **^{1}H-NMR** (300 MHz, $CDCl_3$): δ [ppm] = 4.10 (dd, ^{3}J = 8.2 Hz, ^{3}J = 4.1 Hz, 1H), 0.92 (t, ^{3}J = 7.4 Hz, 3H). **^{13}C-NMR** (75 MHz, $CDCl_3$): δ [ppm] = 147.7, 128.8, 91.1, 78.7, 32.6, 18.2, 13.8. **FT-IR** (neat, ATR): ν [cm^{-1}] = 3394 (br), 2960 (m), 2873 (w), 1685 (s), 1458 (m), 1375 (m), 1265 (m), 1215 (w), 1157 (w), 1083 (m), 1020 (s), 914 (w), 759 (w).

6-Hydroxy-2-ethyl-2*H*-pyran-3(6*H*)-one (**2f**)

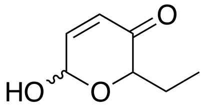

2f

R*f* (cyclohexane/ethyl acetate, 2/1): 0.27. **α-2f**: **^{1}H-NMR** (300 MHz, $CDCl_3$): δ [ppm] = 6.97-6.89 (dd, ^{3}J = 10.4 Hz, ^{3}J = 3.4 Hz, 1H), 6.18-6.10 (d, ^{3}J = 10.3 Hz, 1H), 5.68 (m, 1H), 4.53 (dd, ^{3}J = 7.4 Hz, ^{3}J = 4.1 Hz, 1H), 3.37 (s, 1H), 2.06-1.71 (m, 2H), 1.06-0.97 (m, 3H). **^{13}C-NMR** (75 MHz, $CDCl_3$): δ [ppm] = 196.2, 144.4, 127.7, 87.7, 75.2, 23.0, 23.0, 9.32. **β-2f** (selected signals): **^{1}H-NMR** (300 MHz, $CDCl_3$): δ [ppm] = 4.06-4.02 (m, 1H), 3.65 (dd, ^{3}J = 7.3 Hz, ^{3}J = 4.1 Hz, 1H). **^{13}C-NMR** (75 MHz, $CDCl_3$): δ [ppm] = 147.8, 128.8, 90.9, 80.0, 23.9, 9.6. **FT-IR** (neat, ATR): ν [cm^{-1}] = 3307 (br), 2978 (w), 1666 (s), 1458 (w), 1377 (m), 1267 (m), 1234 (m), 1161 (m), 1112 (m), 1080 (m), 1026 (s), 960 (m), 889 (w), 777 (m), 750 (m), 692 (m).

6-Hydroxy-2-hexyl-2*H*-pyran-3(6*H*)-one (**2g**)

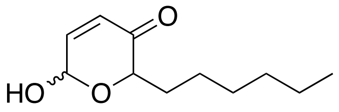

2g

R*f* (cyclohexane/ethyl acetate, 2/1): 0.34. **α-2g**: **^{1}H-NMR** (300 MHz, $CDCl_3$): δ [ppm] = 6.88 (dd, ^3J = 10.2 Hz, ^3J = 3.3 Hz, 1H, H-2), 6.10 (d, ^3J = 10.3 Hz, 1H, H-3), 5.65 (d, ^3J = 3.0 Hz, 1H, H-1), 4.55 (q, ^3J = 3.9, 1H), 3.03 (br s, 1H), 1.95-1.63 (m, 2H), 1.33-1.20 (m, 6H), 0.90-0.85 (m, 3H). **^{13}C-NMR** (75 MHz, $CDCl_3$): δ [ppm] = 196.8, 144.1, 127.7, 87.7, 74.3, 31.7, 29.6, 24.9, 22.6, 14.0. **β-2g** (selected signals): **^{1}H-NMR** (300 MHz, $CDCl_3$): δ [ppm] = 6.94-6.90 (m, 1H), 6.16-6.12 (m, 1H), 5.65 (d, ^3J = 3.0 Hz, 1H), 4.09-4.05 (m, 1H). **^{13}C-NMR** (75 MHz, $CDCl_3$): δ [ppm] = 147.5, 128.8, 90.9, 79.2, 30.7, 29.1, 25.1, 22.8. **FT-IR** (neat, ATR): ν [cm^{-1}] = 3317 (br), 2949 (m), 2873 (m), 1674 (s), 1627 (w), 1469 (m), 1375 (m), 1274 (m), 1242 (m), 1157 (m), 1093 (s), 1026 (s), 999 (m), 952 (m), 891 (m), 785 (m), 700 (m).

(2*S*)-6-Hydroxy-2-chloromethyl-2*H*-pyran-3(6*H*)-one (**2h**)

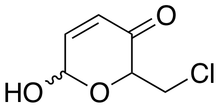

2h

R*f* (cyclohexane/ethyl acetate, 2/1): 0.23. **α-2e**: **^{1}H-NMR** (300 MHz, $CDCl_3$): δ [ppm] = 7.00 (dd, ^3J = 10.3 Hz, ^3J = 3.6 Hz, 1H), 6.19 (d, ^3J = 10.3 Hz, 1H), 5.79 (m, 1H), 4.90 (dd, ^3J = 5.5 Hz, ^3J = 3.2 Hz, 1H), 3.98-3.92 (m, 2H), 3.62-3.58 (m, 1H). **^{13}C-NMR** (75 MHz, $CDCl_3$): δ [ppm] = 193.0, 145.1, 127.5, 87.9, 74.0, 42.9. **β-2e** (selected signals): **^{1}H-NMR** (300 MHz, $CDCl_3$): δ ppm] = 7.03 (dd, ^3J = 10.3 Hz, ^3J = 1.6 Hz, 1H), 6.23 (dd, ^3J = 1.5 Hz, ^3J = 10.3 Hz, 1H), 4.43 (dd, ^3J = 6.3 Hz, ^3J = 3.8 Hz, 1H), 4.01-3.99 (m, 1H). **^{13}C-NMR** (75 MHz, $CDCl_3$): δ [ppm] = 192.6, 148.2, 128.4, 90.4, 78.5, 43.7. **FT-IR** (neat, ATR): ν [cm^{-1}] = 3329 (br), 1666 (s), 1631 (w), 1436 (w), 1371 (w), 1273 (m), 1232 (m), 1192 (w), 1153 (w), 1107 (m), 1080 (m), 1028 (s), 902 (m), 802 (m), 763(m), 731 (m), 690 (m).

6-Hydroxy-2,6-dimethyl-2*H*-pyran-3(6*H*)-one (**2i**)

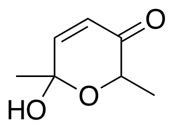

2i

R*f* (cyclohexane/diethyl ether, 2/1): 0.09. **major isomer: ^{1}H-NMR** (300 MHz, $CDCl_3$): δ [ppm]= 6.80 (d, ^3J = 10.2 Hz, 1H), 6.00 (d, ^3J = 10.2 Hz, 1H), 4.64 (q, ^3J = 6.9 Hz, 1H), 2.89 (br s, 1H), 1.63 (s, 3H), 1.36 (d, ^3J = 6.9 Hz, 3H). **^{13}C-NMR** (75 MHz, $CDCl_3$): δ [ppm] = 197.2, 147.9, 126.1, 92.9, 70.7, 28.9, 15.3. **minor isomer** (selected signals): **^{1}H-NMR** (300 MHz, $CDCl_3$): δ [ppm] = 6.86 (d, ^3J = 10.2 Hz, 1H), 6.03 (d, ^3J = 10.2 Hz, 1H), 4.30 (q, ^3J = 6.7 Hz, 1H), 1.61 (s, 3H), 1.44 (d, ^3J = 6.8 Hz, 3H). **^{13}C-NMR** (75 MHz, $CDCl_3$): δ [ppm] = 150.1, 126.0, 74.3, 24.3, 17.3. **FT-IR** (neat, ATR): ν [cm^{-1}] = 3336 (m), 3302 (m), 2987 (w), 1676 (s), 1624 (w), 1444 (m), 1371 (m), 1242 (m), 1145 (m), 1109 (m), 1070 (m), 1028 (s), 939 (m), 902 (m), 842 (m), 688 (m).

6-Hydroxy-2-phenyl-2*H*-pyran-3(6*H*)-one (**2j**)

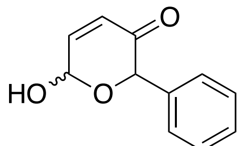

2j

R*f* (cyclohexane/ethyl acetate, 2/1): 0.30. **α-2j**: **^{1}H-NMR** (300 MHz, $CDCl_3$): δ [ppm] = 7.41-7.33 (m, 5H), 6.95 (dd, ^3J = 10.2 Hz, ^3J = 3.3 Hz, 1H), 6.21 (d, ^3J = 10.3 Hz, 1H), 5.73-5.71 (m, 1H), 5.59 (s, 1H), 3.44 (d, ^3J = 5.1 Hz, 1H). **^{13}C-NMR** (75 MHz, $CDCl_3$): δ [ppm] = 194.5, 144.8, 135.2, 128.6, 128.5, 128.0, 127.9, 88.0, 77.0. **β-2j** (selected signals): **^{1}H-NMR** (300 MHz, $CDCl_3$): δ [ppm] = 7.41-7.33 (m, 5H), 7.01-6.97 (m, 1H), 6.26 (dd, ^3J = 10.3 Hz, ^4J = 1.6 Hz, 1H), 5.79-5.76 (m, 1H), 5.09 (d, ^4J = 1.5 Hz, 1H), 3.64 (d, ^3J = 7.2 Hz, 1H). **^{13}C-NMR** (75 MHz, $CDCl_3$): δ [ppm] = 194.1, 148.2, 128.3, 91.5, 81.1. **FT-IR** (neat, ATR): ν [cm^{-1}] = 3392 (br), 1689 (s), 1629 (w), 1469 (w), 1454 (m), 1371 (w), 1290 (m), 1219 (m), 1089 (m), 1074 (m), 1024 (s), 1002 (s), 945 (m), 891 (m), 794 (w), 750 (s), 698 (s).

NMR spectra of the products

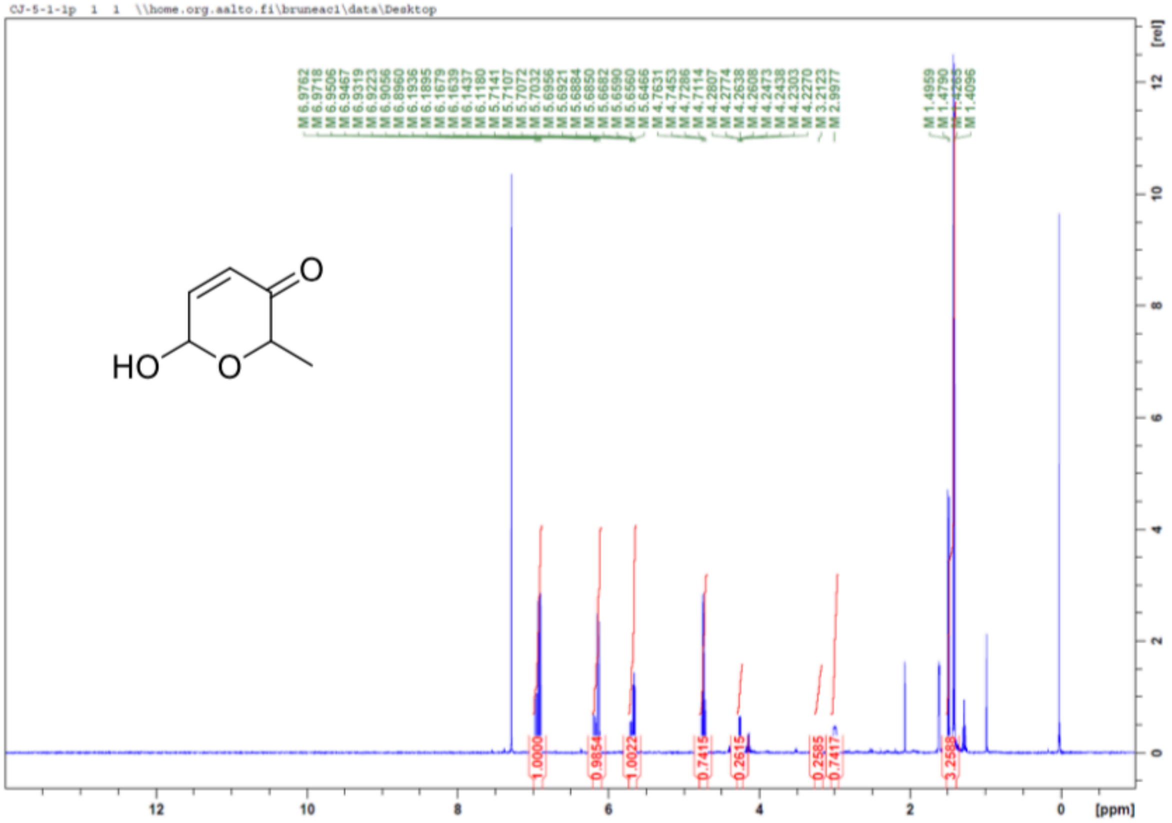

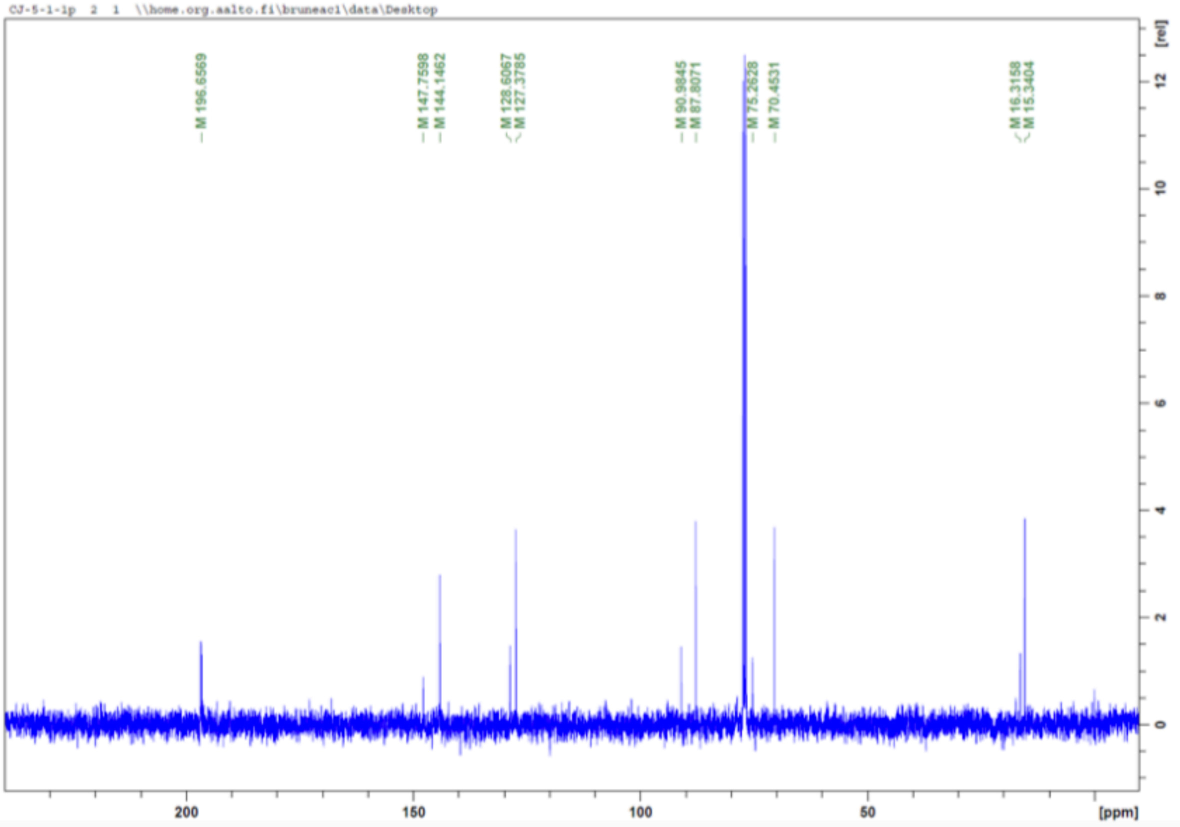

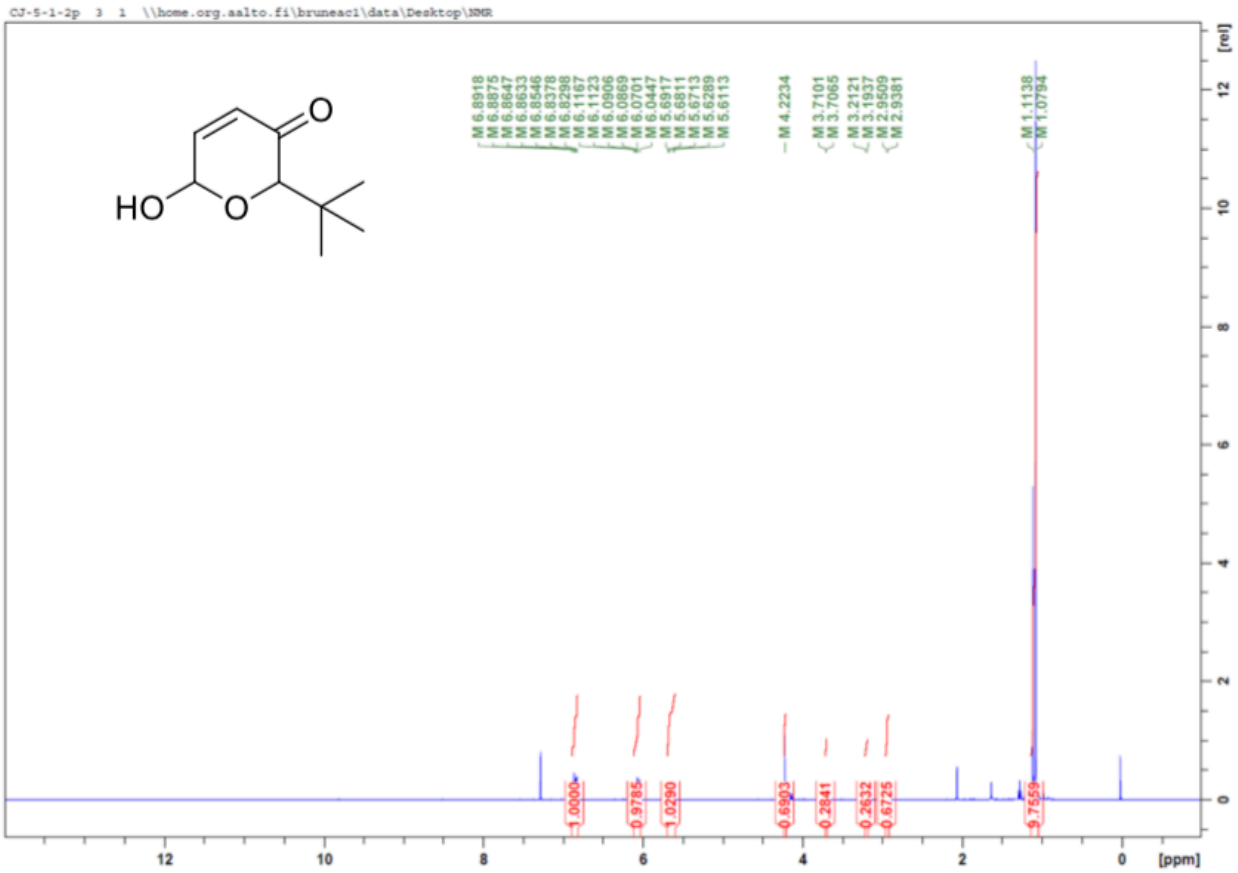
CJ-5-1-2p 3 1 \\home.org.aalto.fi\bruneac1\data\Desktop\NMR
HO
O
O
M 6.8918
M 6.8875
M 6.8647
M 6.8633
M 6.8546
M 6.8378
M 6.8298
M 6.1167
M 6.1123
M 6.0906
M 6.0869
M 6.0701
M 6.0447
M 5.6917
M 5.6811
M 5.6713
M 5.6289
M 5.6113
M 4.2234
M 3.7101
M 3.7065
M 3.2121
M 3.1937
M 2.9509
M 2.9381
M 1.1138
M 1.0794
1.0000
0.9785
1.0290
0.6903
0.2841
0.2632
0.6725
9.7559
[rel]
[ppm]

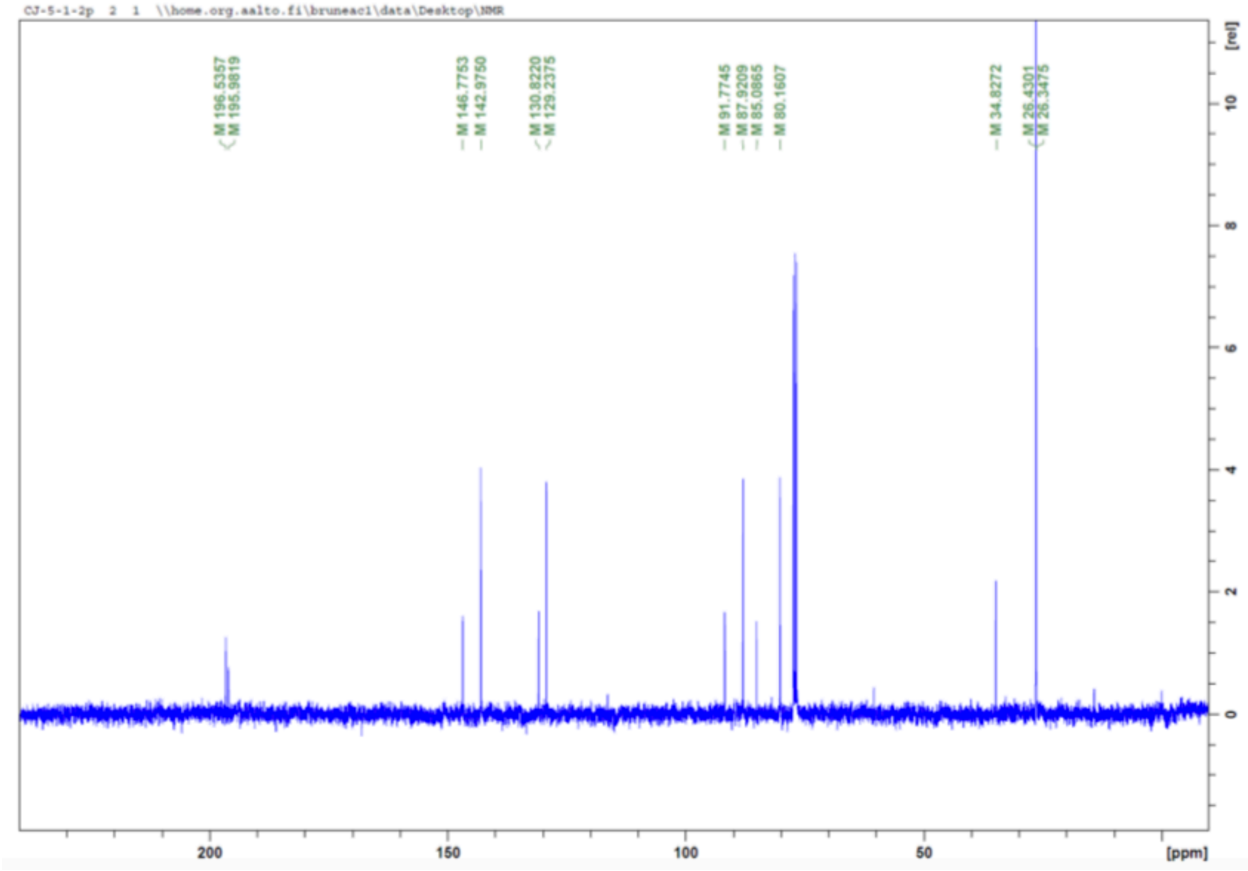
CJ-5-1-2p 2 1 \\home.org.aalto.fi\bruneac1\data\Desktop\NMR
M 196.5357
M 195.9819
M 146.7753
M 142.9750
M 130.8220
M 129.2375
M 91.7745
M 87.9209
M 85.0865
M 80.1607
M 34.8272
M 26.4301
M 26.3475
[rel]
[ppm]

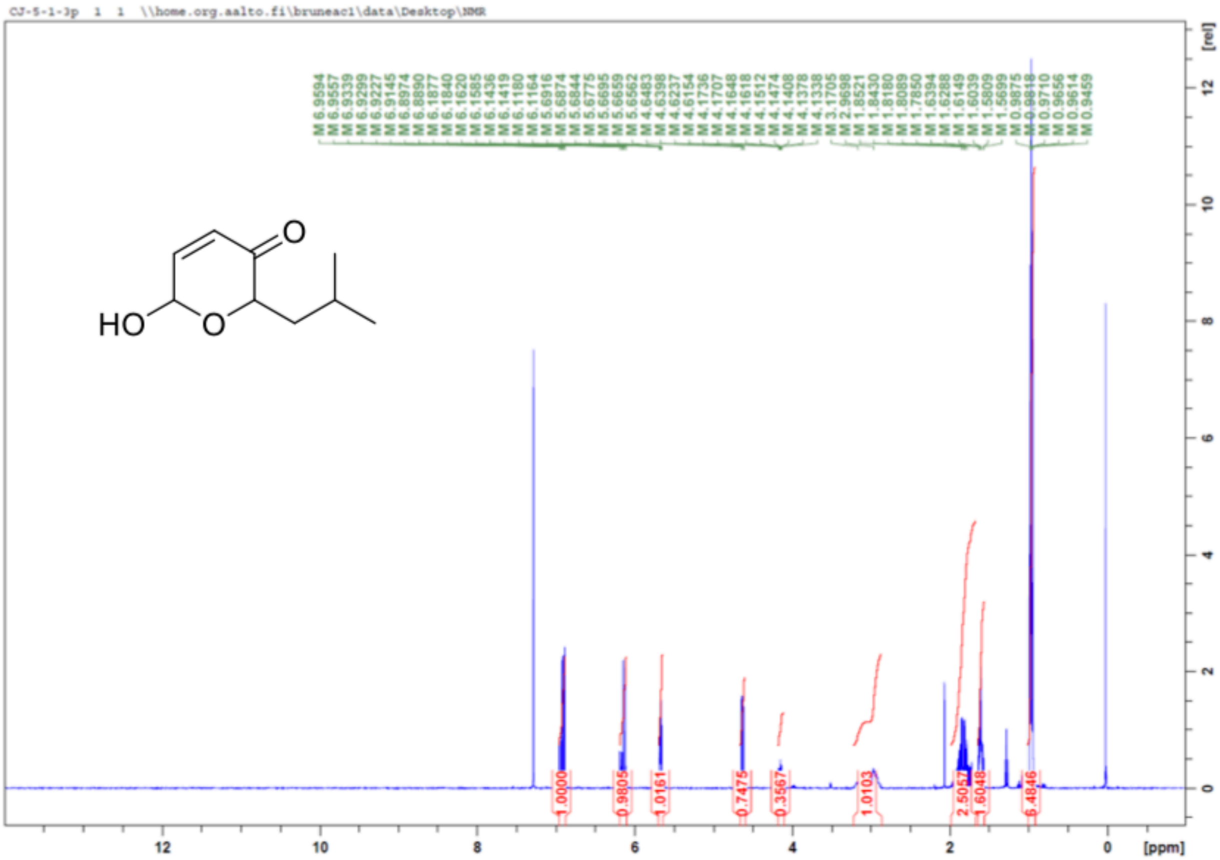
CJ-5-1-3p 1 1 \\home.org.aalto.fi\bruneac1\data\Desktop\NMR
M 6.9594
M 6.9557
M 6.9339
M 6.9299
M 6.9227
M 6.9145
M 6.8974
M 6.8890
M 6.1877
M 6.1840
M 6.1620
M 6.1585
M 6.1436
M 6.1419
M 6.1180
M 6.1164
M 5.6916
M 5.6874
M 5.6844
M 5.6775
M 5.6695
M 5.6659
M 5.6562
M 4.6483
M 4.6398
M 4.6237
M 4.6154
M 4.1736
M 4.1707
M 4.1648
M 4.1618
M 4.1512
M 4.1474
M 4.1408
M 4.1378
M 4.1338
M 3.1705
M 2.9698
M 1.8521
M 1.8430
M 1.8180
M 1.8089
M 1.7850
M 1.6394
M 1.6288
M 1.6149
M 1.6039
M 1.5809
M 1.5699
M 0.9875
M 0.9818
M 0.9710
M 0.9656
M 0.9614
M 0.9459
HO
O
O
1.0000
0.9805
1.0161
0.7475
0.3567
1.0103
2.5057
1.6048
6.4846
[ppm]
[rel]

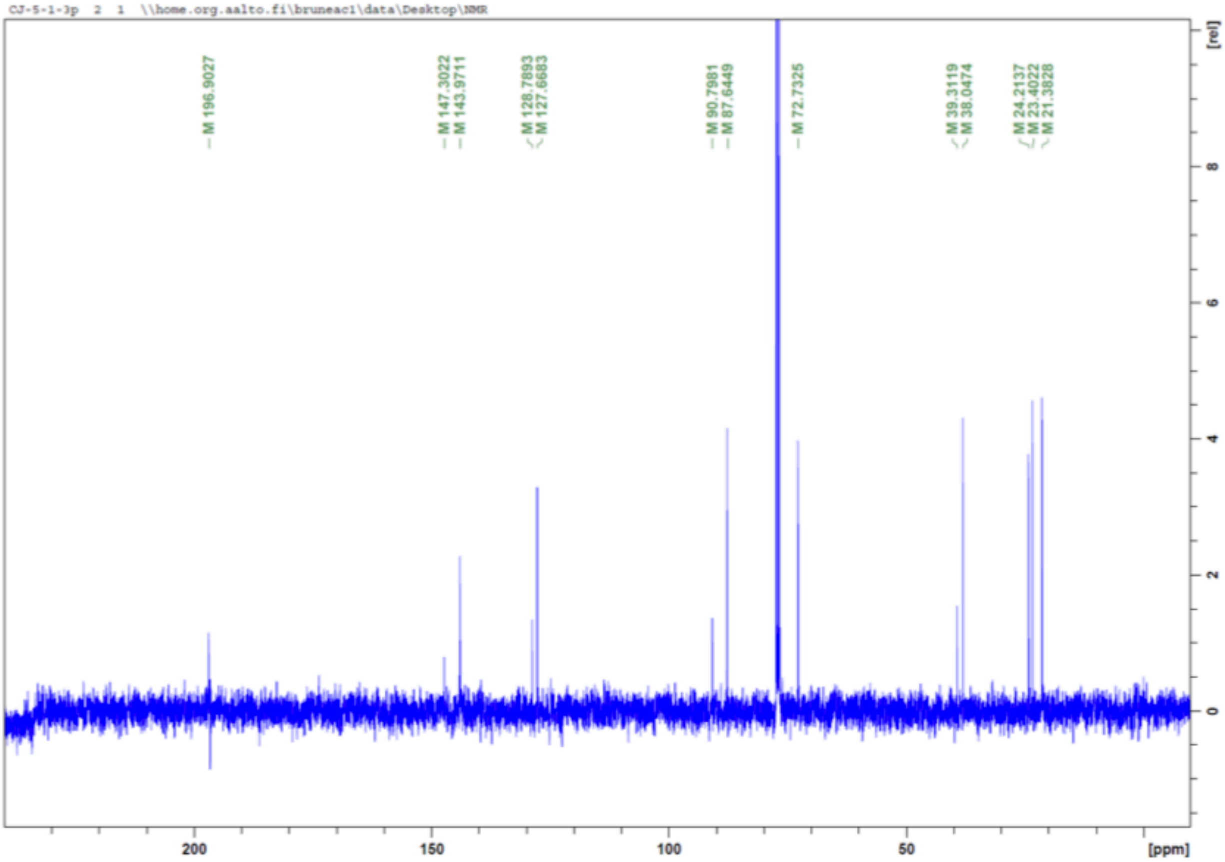
CJ-5-1-3p 2 1 \\home.org.aalto.fi\bruneac1\data\Desktop\NMR
M 196.9027
M 147.3022
M 143.9711
M 128.7893
M 127.6683
M 90.7981
M 87.6449
M 72.7325
M 39.3119
M 38.0474
M 24.2137
M 23.4022
M 21.3828
[ppm]
[rel]

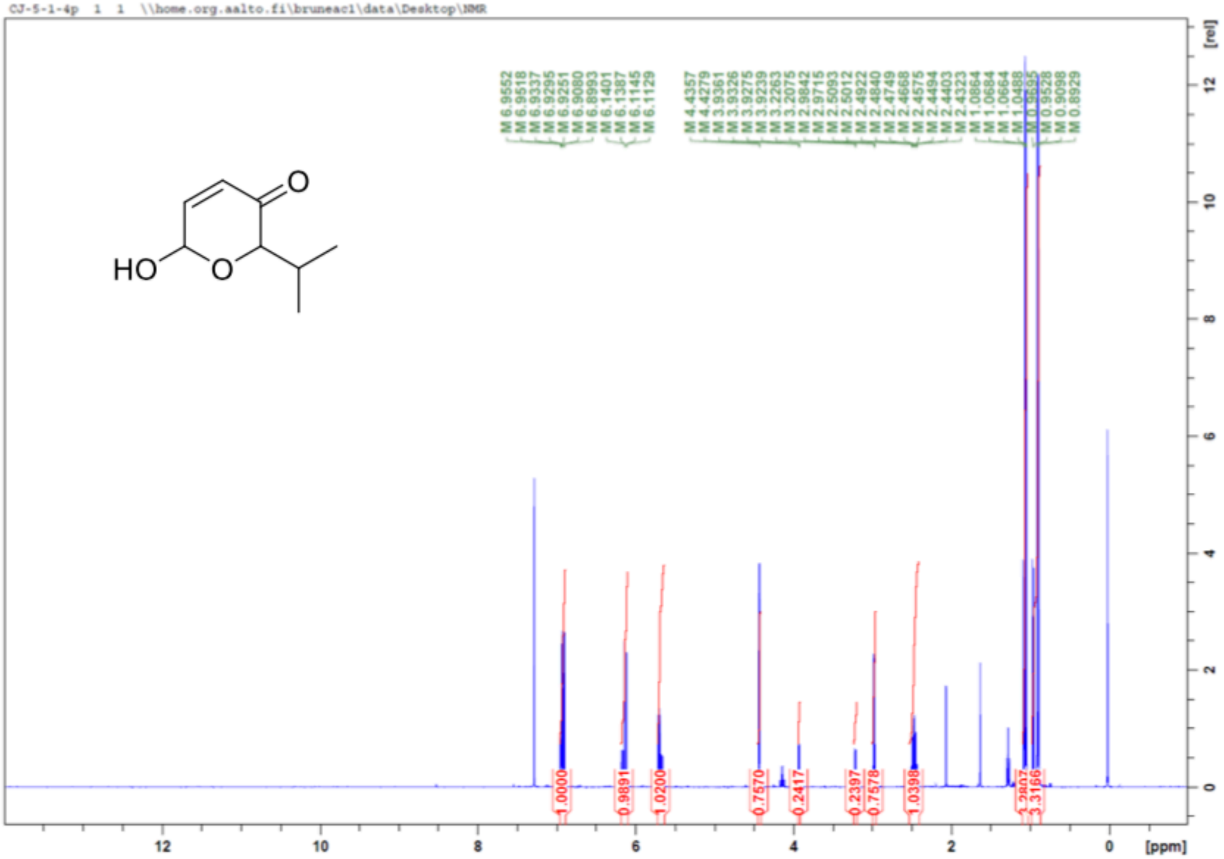
CJ-5-1-4p 1 1 \\home.org.aalto.fi\bruneac1\data\Desktop\NMR
HO
O
O
M 6.9552
M 6.9518
M 6.9337
M 6.9295
M 6.9251
M 6.9080
M 6.8993
M 6.1401
M 6.1387
M 6.1145
M 6.1129
M 4.4357
M 4.4279
M 3.9361
M 3.9326
M 3.9275
M 3.9239
M 3.2263
M 3.2075
M 2.9842
M 2.9715
M 2.5093
M 2.5012
M 2.4922
M 2.4840
M 2.4749
M 2.4668
M 2.4575
M 2.4494
M 2.4403
M 2.4323
M 1.0864
M 1.0664
M 1.0488
M 0.9528
M 0.9098
M 0.8929
1.0000
0.9891
1.0200
0.7570
0.2417
0.2397
0.7578
1.0398
3.2807
3.3166
[rel]
[ppm]

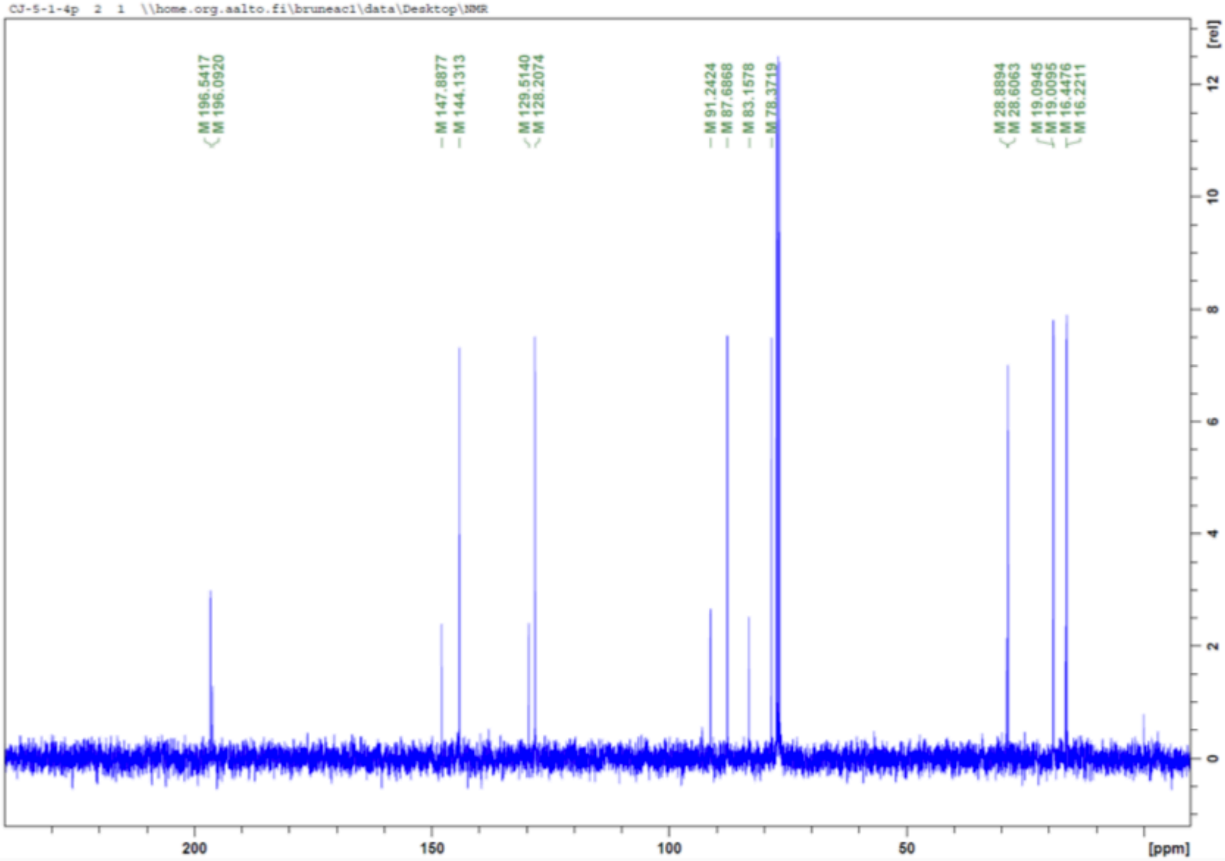
CJ-5-1-4p 2 1 \\home.org.aalto.fi\bruneac1\data\Desktop\NMR
M 196.5417
M 196.0920
M 147.8877
M 144.1313
M 129.5140
M 128.2074
M 91.2424
M 87.6868
M 83.1578
M 78.3719
M 28.8894
M 28.6063
M 19.0945
M 19.0095
M 16.4476
M 16.2211
[rel]
[ppm]

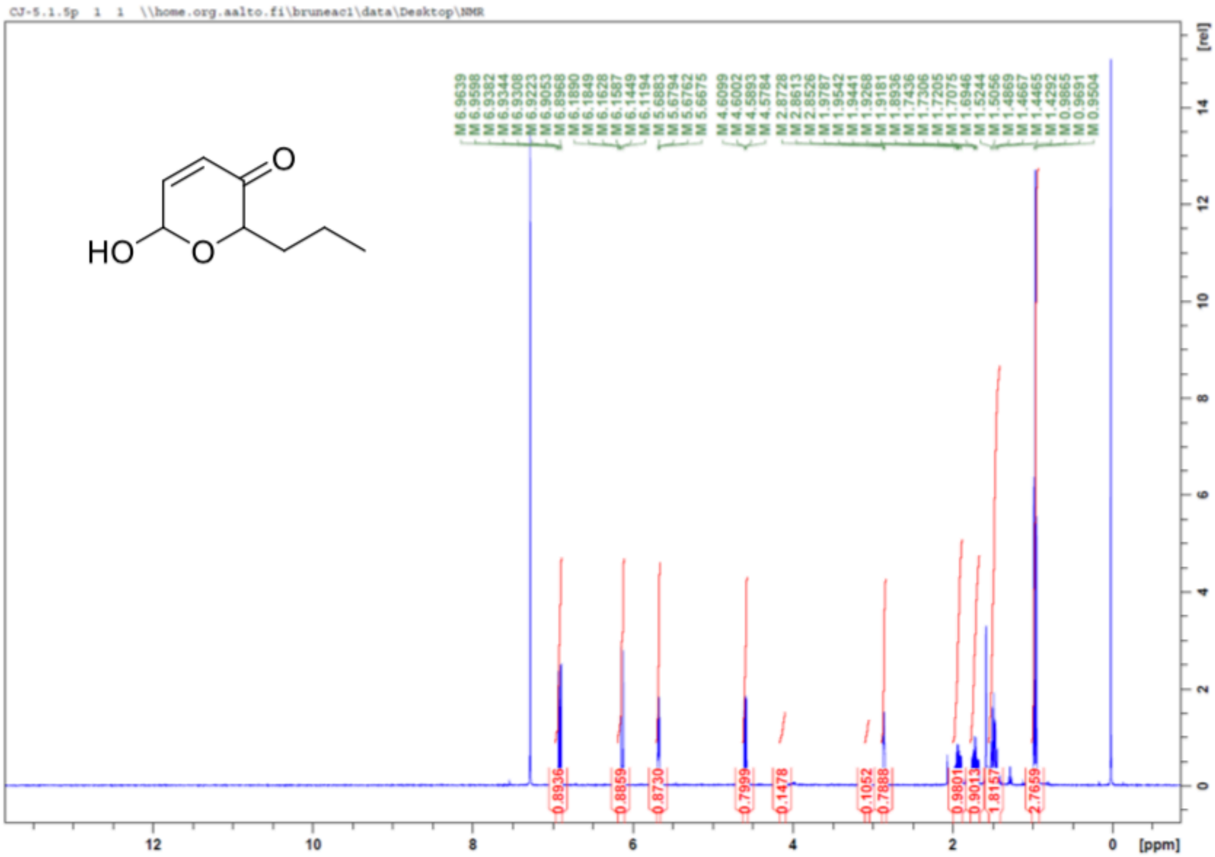
CJ-5.1.5p 1 1 \\home.org.aalto.fi\bruneac1\data\Desktop\NMR
M 6.9639
M 6.9598
M 6.9382
M 6.9344
M 6.9308
M 6.9223
M 6.9053
M 6.8968
M 6.1890
M 6.1849
M 6.1628
M 6.1587
M 6.1449
M 6.1194
M 5.6883
M 5.6794
M 5.6762
M 5.6675
M 4.6099
M 4.6002
M 4.5893
M 4.5784
M 2.8728
M 2.8613
M 2.8526
M 1.9787
M 1.9542
M 1.9441
M 1.9268
M 1.9181
M 1.8936
M 1.7436
M 1.7306
M 1.7205
M 1.7075
M 1.6946
M 1.5244
M 1.5056
M 1.4869
M 1.4667
M 1.4465
M 1.4292
M 0.9865
M 0.9691
M 0.9504
HO
O
O
0.8936
0.8859
0.8730
0.7999
0.1478
0.1052
0.7888
0.9801
0.9013
1.8157
2.7659
[rel]
[ppm]

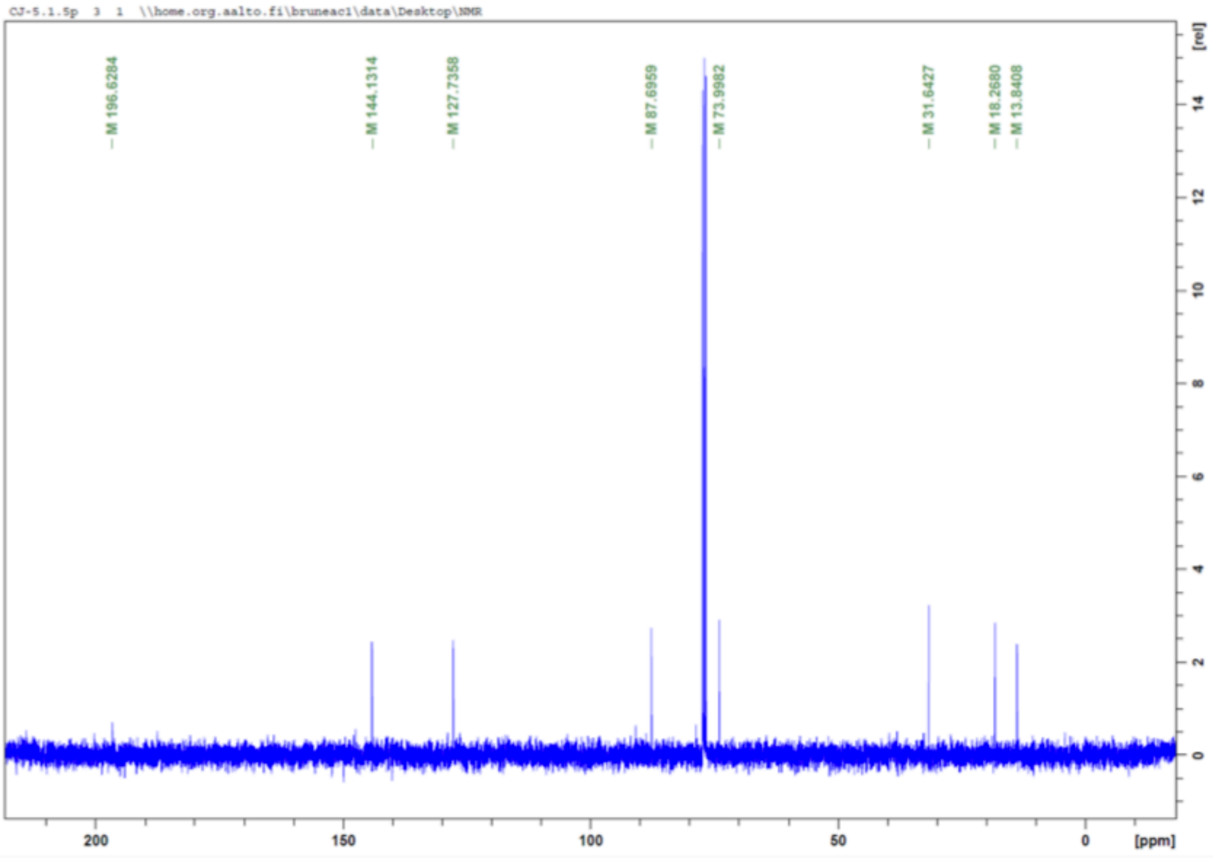
CJ-5.1.5p 3 1 \\home.org.aalto.fi\bruneac1\data\Desktop\NMR
M 196.6284
M 144.1314
M 127.7358
M 87.6959
M 73.9982
M 31.6427
M 18.2680
M 13.8408
[rel]
[ppm]

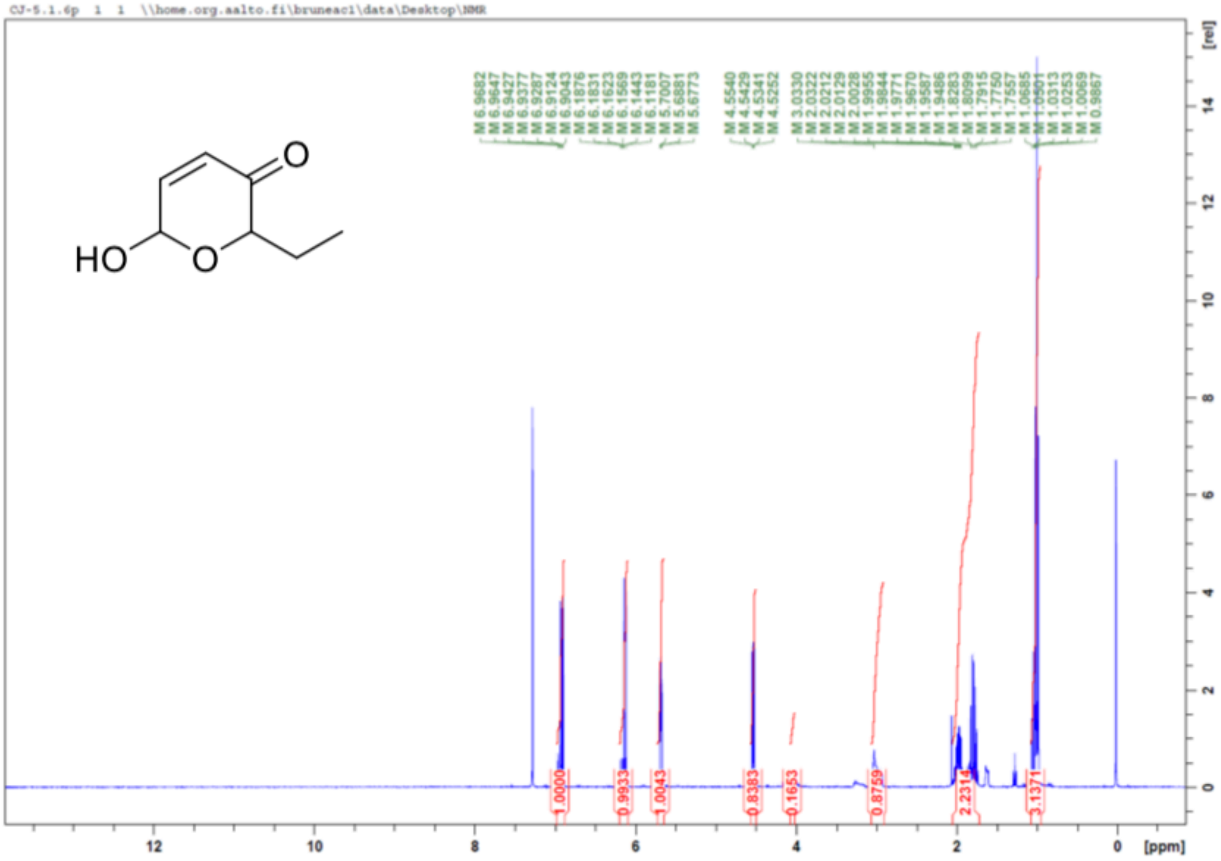
CJ-5.1.6p 1 1 \\home.org.aalto.fi\bruneac1\data\Desktop\NMR
HO
O
O
M 6.9682
M 6.9647
M 6.9427
M 6.9377
M 6.9287
M 6.9124
M 6.9043
M 6.1876
M 6.1831
M 6.1623
M 6.1569
M 6.1443
M 6.1181
M 5.7007
M 5.6881
M 5.6773
M 4.5540
M 4.5429
M 4.5341
M 4.5252
M 3.0330
M 2.0322
M 2.0212
M 2.0129
M 2.0028
M 1.9955
M 1.9844
M 1.9771
M 1.9587
M 1.9486
M 1.8283
M 1.8099
M 1.7915
M 1.7750
M 1.7557
M 1.0685
M 1.0501
M 1.0313
M 1.0253
M 1.0069
M 0.9867
1.0000
0.9933
1.0043
0.8383
0.1653
0.8759
2.2314
3.1371
[rel]
[ppm]

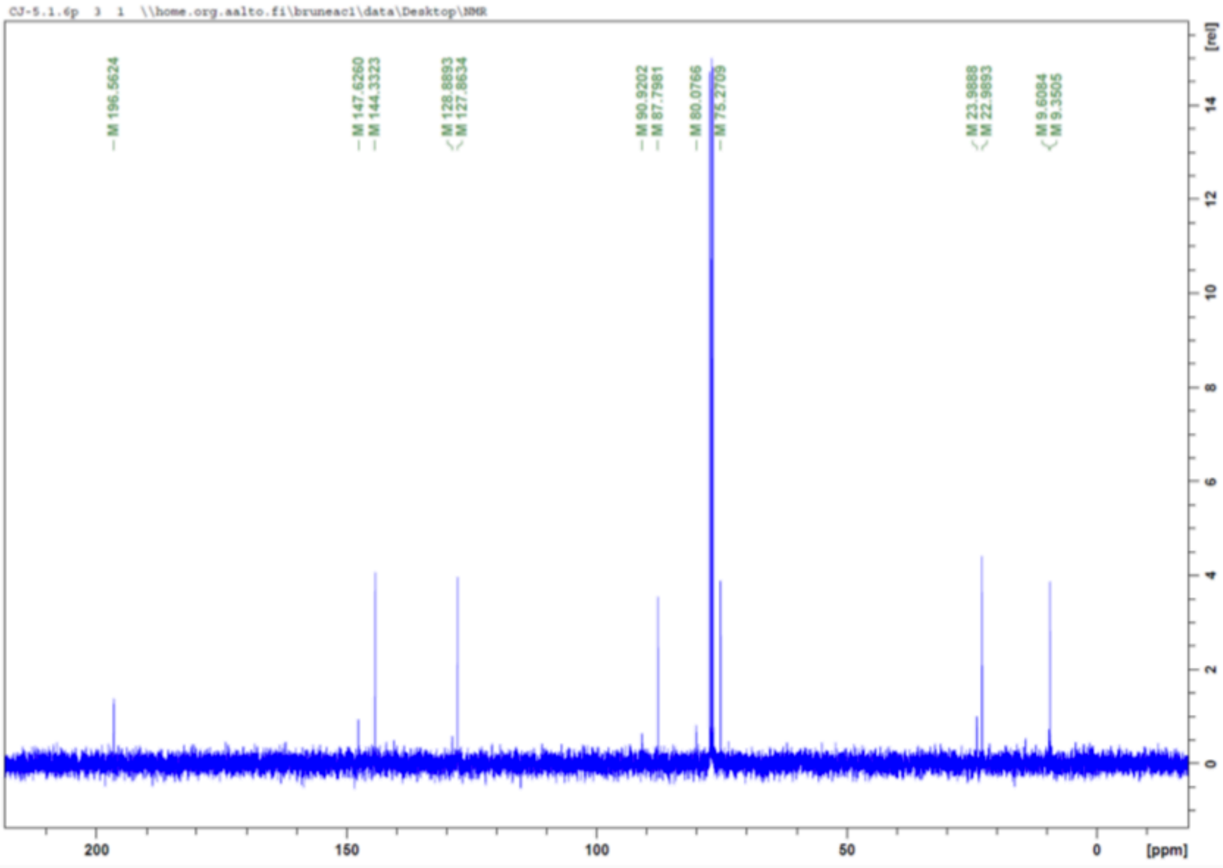
CJ-5.1.6p 3 1 \\home.org.aalto.fi\bruneac1\data\Desktop\NMR
M 196.5624
M 147.6260
M 144.3323
M 128.8893
M 127.8634
M 90.9202
M 87.7981
M 80.0766
M 75.2709
M 23.9888
M 22.9893
M 9.6084
M 9.3505
[rel]
[ppm]

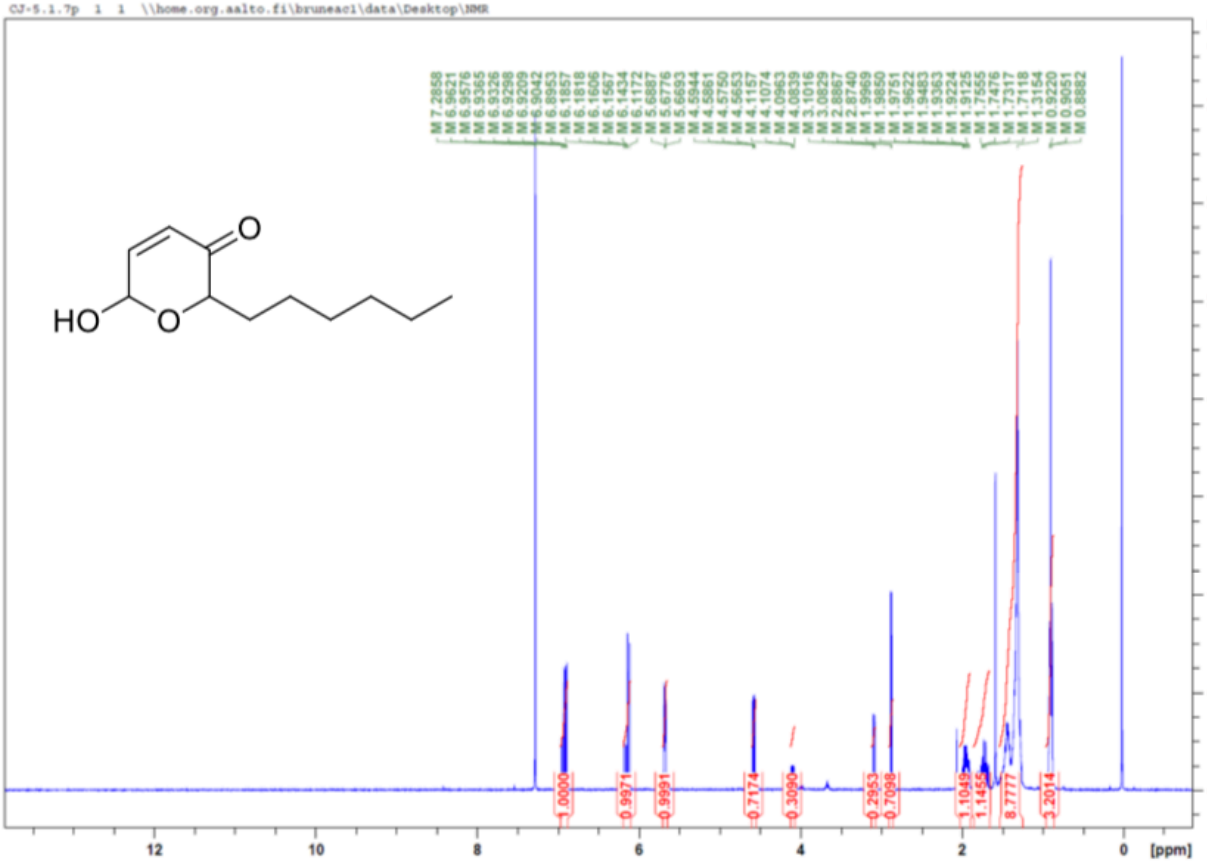
CJ-5.1.7p 1 1 \\home.org.aalto.fi\bruneac1\data\Desktop\NMR
HO
O
O
M 7.2858
M 6.9621
M 6.9576
M 6.9365
M 6.9326
M 6.9298
M 6.9209
M 6.9042
M 6.8953
M 6.1857
M 6.1818
M 6.1606
M 6.1567
M 6.1434
M 6.1172
M 5.6887
M 5.6776
M 5.6693
M 4.5944
M 4.5861
M 4.5750
M 4.5653
M 4.1157
M 4.1074
M 4.0963
M 4.0839
M 3.1016
M 3.0829
M 2.8867
M 2.8740
M 1.9969
M 1.9850
M 1.9751
M 1.9622
M 1.9483
M 1.9363
M 1.9224
M 1.9125
M 1.7555
M 1.7476
M 1.7317
M 1.7118
M 1.3154
M 0.9220
M 0.9051
M 0.8882
1.0000
0.9971
0.9991
0.7174
0.3090
0.2953
0.7098
1.1049
1.1455
8.7777
3.2014
12
10
8
6
4
2
0
[ppm]
[rel]
14

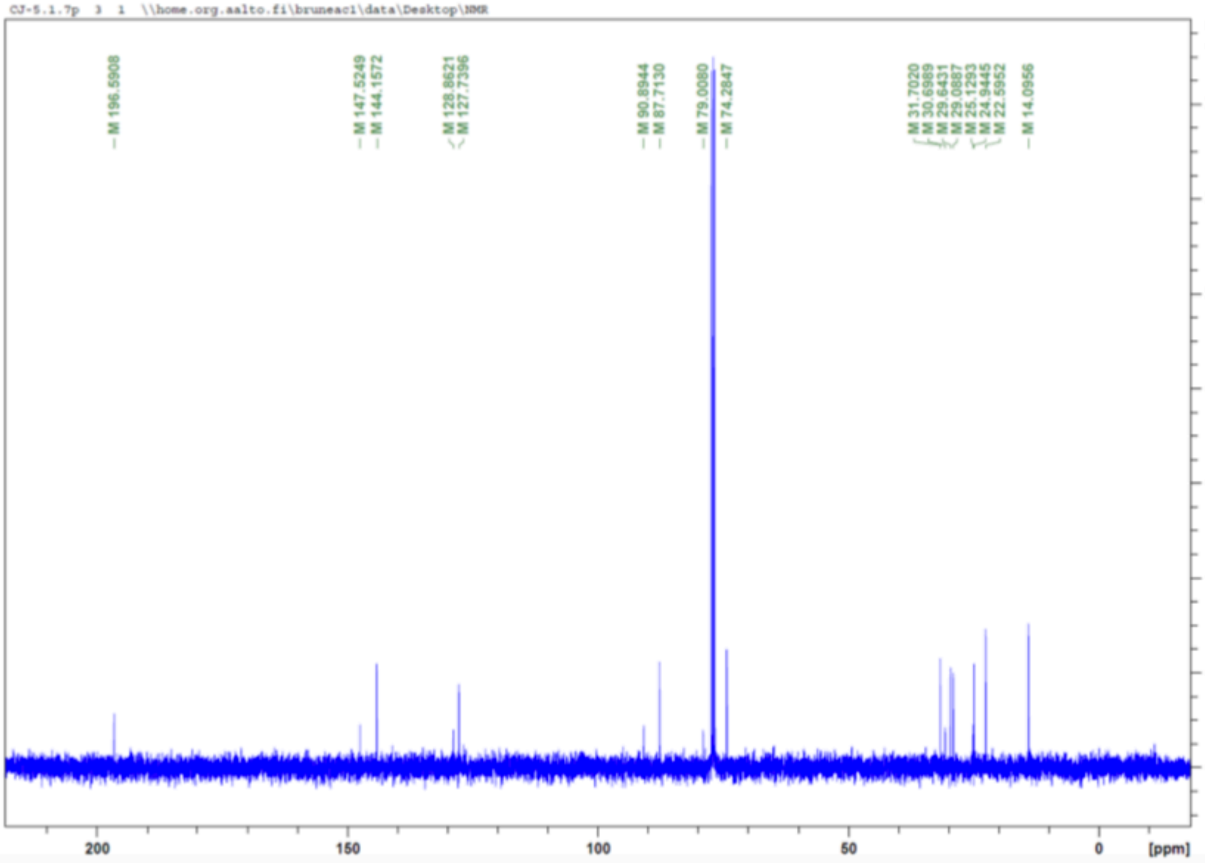
CJ-5.1.7p 3 1 \\home.org.aalto.fi\bruneac1\data\Desktop\NMR
M 196.5908
M 147.5249
M 144.1572
M 128.8621
M 127.7396
M 90.8944
M 87.7130
M 79.0080
M 74.2847
M 31.7020
M 30.6989
M 29.6431
M 29.0887
M 25.1293
M 24.9445
M 22.5952
M 14.0956
200
150
100
50
0
[ppm]
[rel]

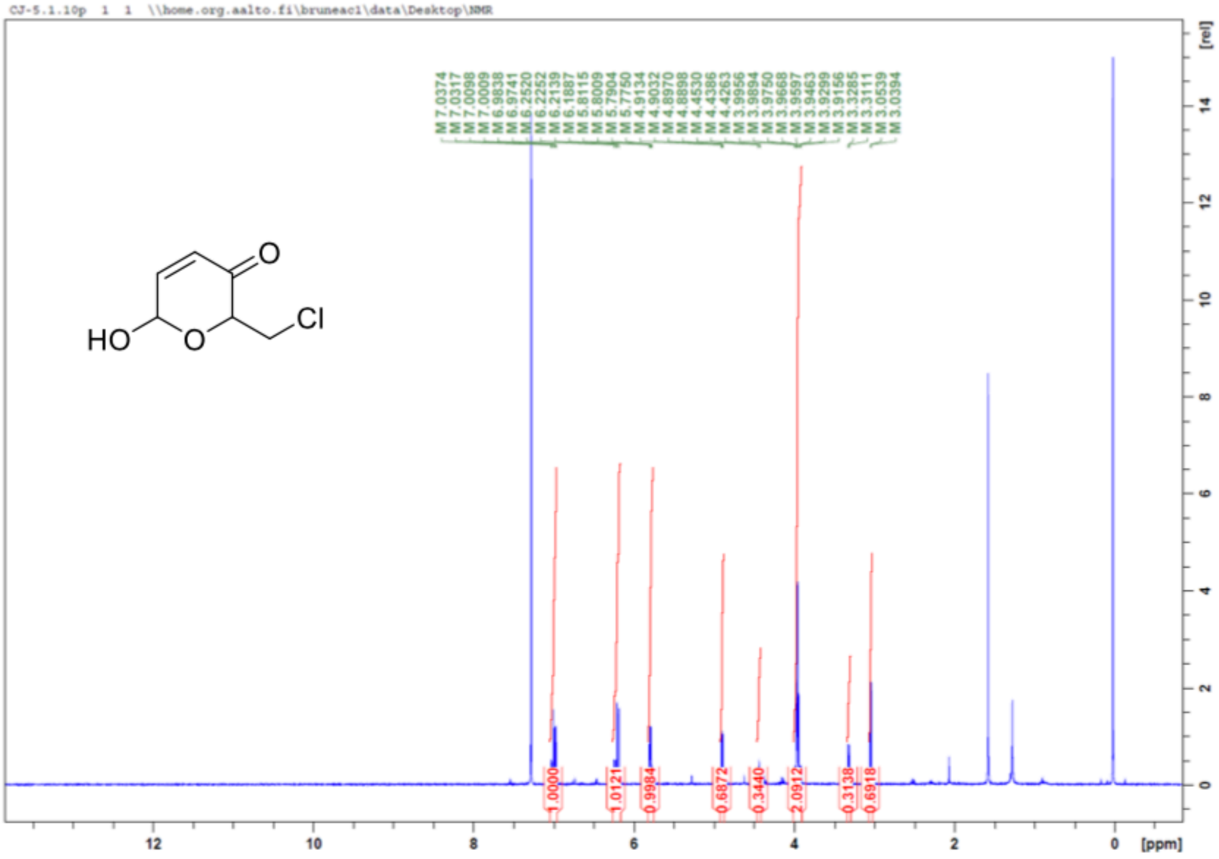

CJ-5.1.10p 1 1 \\home.org.aalto.fi\bruneac1\data\Desktop\NMR
M 7.0374
M 7.0317
M 7.0098
M 7.0009
M 6.9838
M 6.9741
M 6.2520
M 6.2252
M 6.2139
M 6.1887
M 5.8115
M 5.8009
M 5.7904
M 5.7750
M 4.9134
M 4.9032
M 4.8970
M 4.8898
M 4.4530
M 4.4386
M 4.4263
M 3.9956
M 3.9894
M 3.9750
M 3.9668
M 3.9597
M 3.9463
M 3.9299
M 3.9156
M 3.3285
M 3.3111
M 3.0539
M 3.0394
1.0000
1.0121
0.9984
0.6872
0.3440
2.0912
0.3138
0.6918
HO
O
O
Cl
[ppm]
[rel]

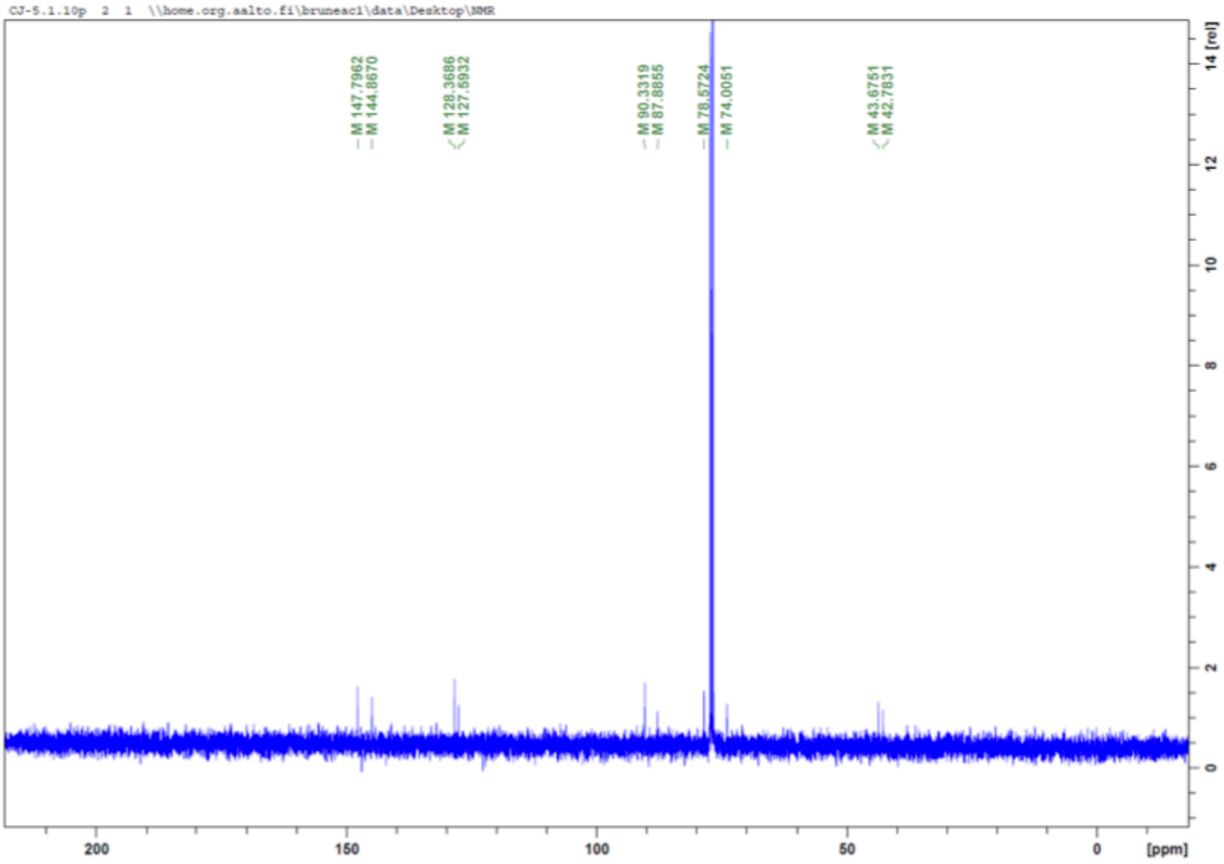

CJ-5.1.10p 2 1 \\home.org.aalto.fi\bruneac1\data\Desktop\NMR
M 147.7962
M 144.8670
M 128.3686
M 127.5932
M 90.3319
M 87.8855
M 78.5724
M 74.0051
M 43.6751
M 42.7831
[ppm]
[rel]

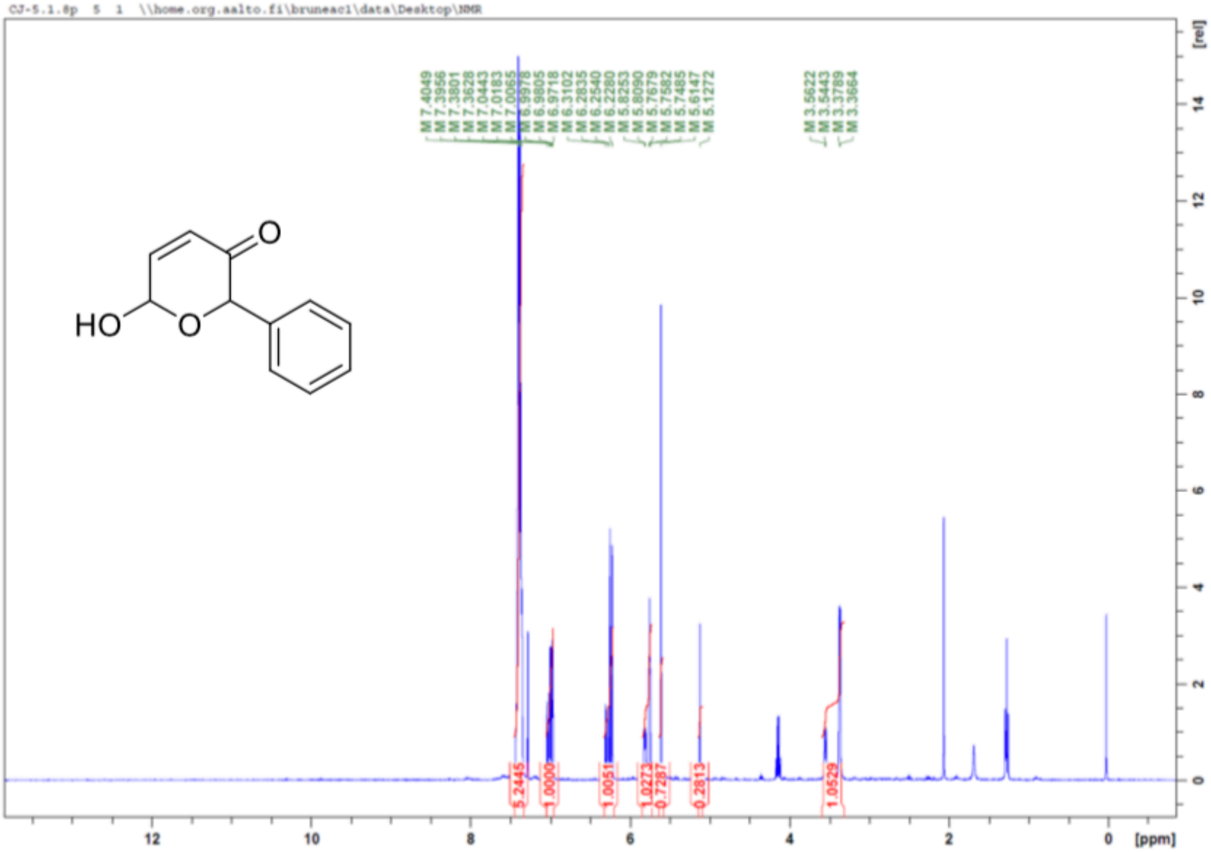
CJ-5.1.8p 5 1 \\home.org.aalto.fi\bruneac1\data\Desktop\NMR
M 7.4049
M 7.3956
M 7.3801
M 7.3628
M 7.0443
M 7.0183
M 7.0065
M 6.9978
M 6.9805
M 6.9718
M 6.3102
M 6.2835
M 6.2540
M 6.2280
M 5.8253
M 5.8090
M 5.7679
M 5.7582
M 5.7485
M 5.6147
M 5.1272
M 3.5622
M 3.5443
M 3.3789
M 3.3664
HO
O
O
5.2445
1.0000
1.0051
1.0273
0.7287
0.2813
1.0529
[ppm]
[rel]

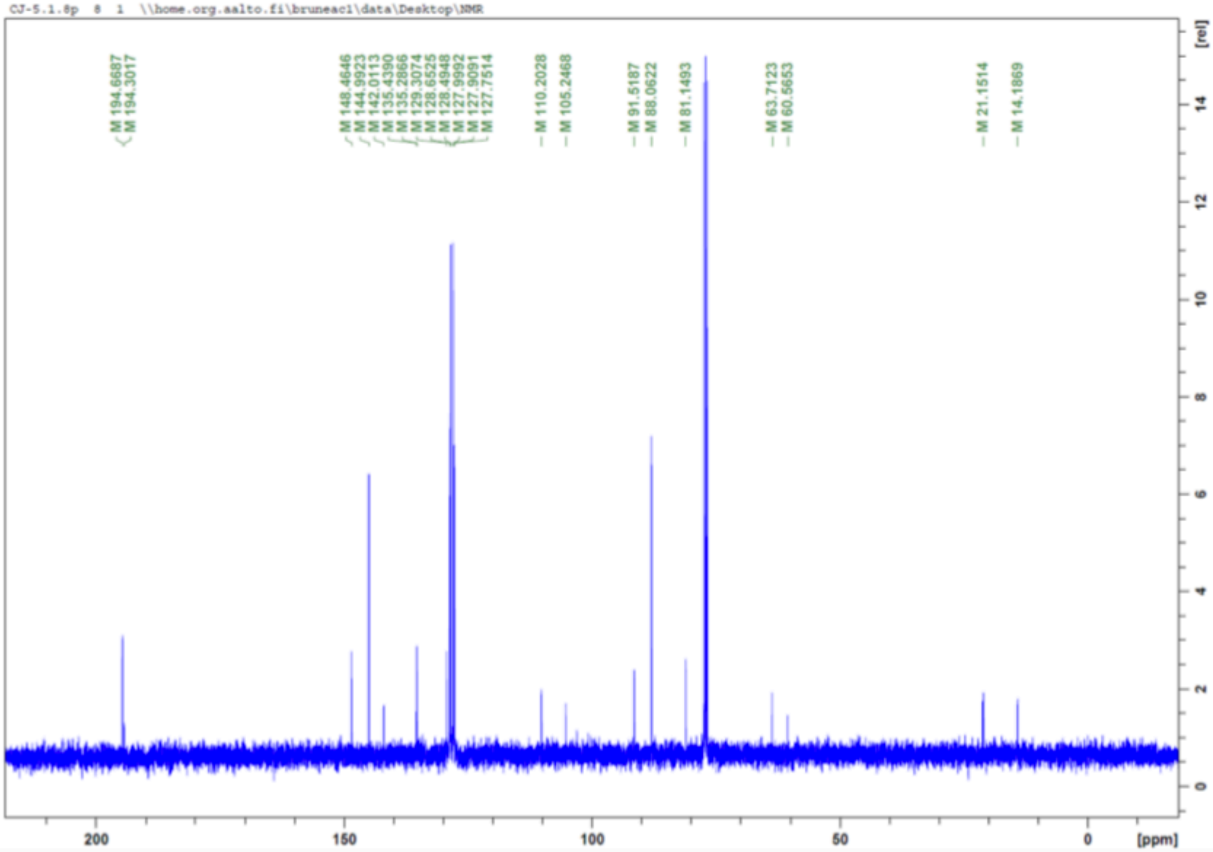
CJ-5.1.8p 8 1 \\home.org.aalto.fi\bruneac1\data\Desktop\NMR
M 194.6687
M 194.3017
M 148.4646
M 144.9923
M 142.0113
M 135.4390
M 135.2866
M 129.3074
M 128.6525
M 128.4948
M 127.9992
M 127.9091
M 127.7514
M 110.2028
M 105.2468
M 91.5187
M 88.0622
M 81.1493
M 63.7123
M 60.5653
M 21.1514
M 14.1869
[ppm]
[rel]
